# Monolithic Integration of Redox-Stable Sn–Pb Halide Perovskite Single-Crystalline Films for Durable Near-Infrared Photodetection

**DOI:** 10.1007/s40820-025-01991-y

**Published:** 2026-01-12

**Authors:** Rajendra Kumar Gunasekaran, Jihoon Nam, Myeong-geun Choi, Won Chang Choi, Sunwoo Kim, Doyun Im, Yeonghun Yun, Yun Hwa Hong, Sang Hyeok Ryou, Hyungwoo Lee, Kwang Heo, Sangwook Lee

**Affiliations:** 1 School of Materials Science and Engineering, Kyungpook National University (KNU), Daegu, 41566 Republic of Korea; 2 School of Physics, The University of Sydney, NSW, 2006 Australia; 3 The University of Sydney Nano Institute (Sydney Nano), The University of Sydney, NSW, 2006 Australia; 4 Department Perovskite Tandem Solar Cells, Helmholtz-Zentrum Berlin Für Materialien Und Energie GmbH, 12489 Berlin, Germany; 5 Department of Nanotechnology and Advanced Materials Engineering, Hybrid Materials Research Center (HMC), Sejong University (SJU), Seoul, 05006 Republic of Korea; 6 Department of Physics, Department of Energy Systems Research, Ajou University, Suwon, 16499 Republic of Korea

**Keywords:** Tin–lead perovskite, Near-infrared photodetectors, Single-crystal thin films, Coordination chemistry, Low-temperature crystallization

## Abstract

Tin–lead (Sn–Pb) halide perovskite single crystals combine narrow bandgaps, long carrier diffusion lengths, and low trap densities, positioning them as ideal candidates for near-infrared (NIR) optoelectronics. However, conventional growth strategies rely on bulk crystallization at elevated temperatures, leading to uncontrolled nucleation, Sn^2+^ oxidation, and poor compatibility with planar integration. Here, we develop a coordination-engineered crystallization strategy that enables direct, low-temperature growth of micrometer-thick Sn–Pb single-crystal thin films on device-compatible substrates. By modulating metal–solvent coordination strength using a low-donor number cosolvent system, we delineate a narrow processing window that stabilizes precursor speciation, lowers the nucleation barrier, and guides directional crystal growth under mild thermal conditions (< 40 °C). The resulting crystal films exhibit smooth morphology, high crystallinity, compositional uniformity, and ultralow trap densities (~ 3.98 × 10^12^ cm^−3^). When integrated into NIR photodetectors, these films deliver high responsivity (0.51 A W^−1^ at 900 nm), specific detectivity up to 3.6 × 10^12^ Jones, fast response (~ 188 μs), and > 25,000 cycles of ambient operational stability. This approach establishes a scalable platform for redox-stable, low-temperature growth of Sn–Pb perovskite crystal films and expands the processing–structure–function landscape for next-generation infrared optoelectronics.

**Supplementary Information:**

The online version contains supplementary material available at 10.1007/s40820-025-01991-y.

## Highlights

- Cosolvent-coordinated crystallization at ≤40 °C enables planar integration of micrometer-thick Sn–Pb single-crystal films with high structural and composition integrity.
- A tailored solvent matrix yields thickness-tunable single-crystal thin films with ultralow trap densities (~3.98 × 10^12^ cm^−3^) and robust ambient stability.
- Integrated near-infrared photodetectors achieve 73.8% EQE, 0.51 A W^−1^ responsivity, 3.6 × 10^12^ Jones specific detectivity, and stable performance over 25,000 cycles.

**Supplementary Information:**

The online version contains supplementary material available at 10.1007/s40820-025-01991-y.

## Graphical abstract

**Figure:**
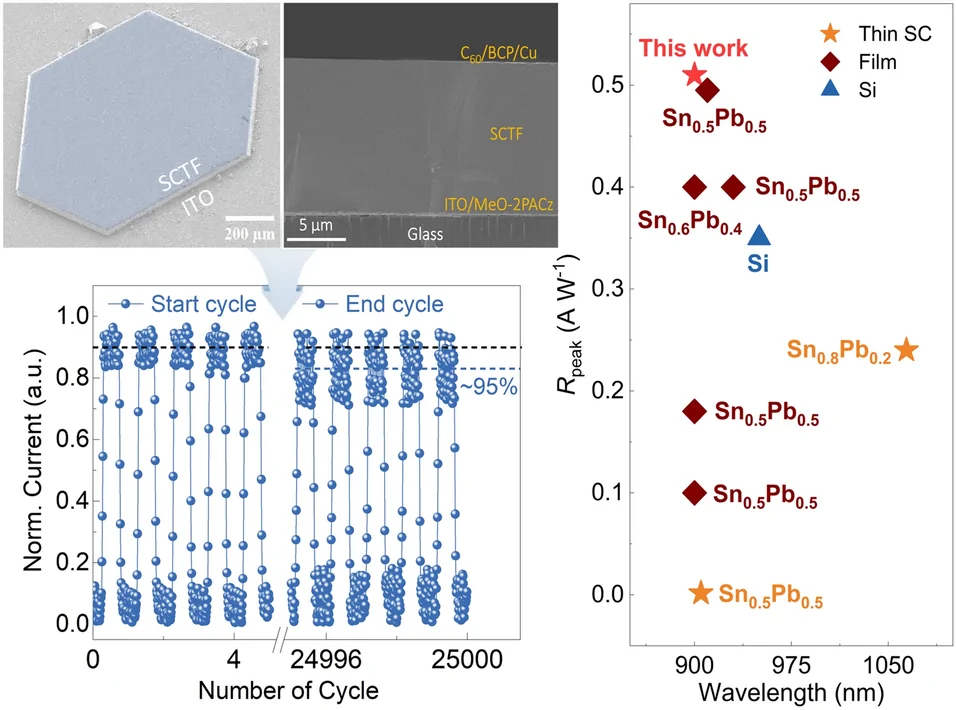

## Introduction

Near-infrared (NIR) optoelectronics are pivotal to advancing energy-efficient sensing, biomedical imaging, and optical communication [1, 2], yet remain constrained by the lack of scalable semiconductors that combine deep NIR absorption with ambient stability and low-temperature processability [3, 4]. Crystalline silicon and InGaAs offer excellent NIR responsivity but are constrained by high cost, mechanical rigidity, and limited compatibility with solution-based fabrication [5, 6]. Metal halide perovskites offer an attractive alternative, with tunable bandgaps, long carrier diffusion lengths, and intrinsic solution processability [7, 8]. Tin–lead (Sn–Pb) halide perovskites are among the few solution-processable semiconductors capable of accessing bandgaps near 1.2 eV, with high absorption coefficients and intrinsic carrier mobilities well suited for infrared detection [7, 8]. However, integrating Sn–Pb perovskites into practical planar devices remains fundamentally limited by their redox sensitivity and structural inhomogeneity [11]. Polycrystalline films suffer from grain-boundary recombination, phase segregation, and rapid Sn^2+^ oxidation, while surface passivation offers only incremental improvements to a defect-rich microstructure [14, 15].

Single-crystal thin films (SCTFs) offer a structurally coherent, trap-suppressed alternative, but scalable low-temperature growth compatible with planar device integration remains elusive [16, 17]. Recent efforts to grow Sn–Pb SCTFs have highlighted a narrow and poorly defined processing window. For instance, MAPb_0.5_Sn_0.5_I_3_ crystal films grown via inverse-temperature crystallization at ~ 95 °C exhibited high thickness and crystallinity, but required thermal conditions that accelerate Sn^2+^ oxidation and limit integration with temperature-sensitive substrates [18]. Attempts to reduce processing temperatures through Sn-deficient formulations have led to bandgap broadening (~ 1.35 eV), compromising NIR absorption [19]. Together, these studies highlight a core limitation in Sn–Pb crystallization: the lack of a chemically defined processing window that enables nucleation and crystal growth without triggering Sn^2+^ oxidation or compromising bandgap integrity [20, 21].

To reconcile redox stability with NIR sensitivity, we adopt a multicomponent A-site (FA/MA/Cs) design. Mixed A-site cations (i) confer entropic stabilization that broadens the single-crystal growth window [22], (ii) tune the tolerance factor to suppress octahedral tilts and stabilize the 3D perovskite [23], (iii) improve defect chemistry—lowering the propensity for Sn^2+^oxidation and vacancy formation [24], and (iv) permit bandgap targeting near ~ 1.26 eV for NIR detection [25]. Guided by our prior FA/MA/Cs compositional mapping [26], which identified stability windows associated with reduced defect density and stronger device metrics, we use FA_0.55_MA_0.40_Cs_0.05_, which lies in this window and complements the coordination-modulated crystallization employed here.

Here, we address this challenge by developing a coordination-modulated crystallization strategy, using solvent–metal interactions as a tunable parameter to define a redox-stable growth window. Guided by the Gutmann donor number as a quantitative measure of solvent Lewis basicity, we identify a low-donor cosolvent system—γ-butyrolactone (GBL) and propylene carbonate (PC)—that moderates coordination strength to stabilize halide-rich precursor complexes, suppress Sn^2+^ oxidation, and enable directional nucleation below 40 °C. This coordination-guided strategy allows direct growth of micrometer-thick, planar Sn–Pb SCTFs on functional substrates without high thermal conditions or additive passivation. The resulting films exhibit smooth morphology, high crystallinity, ultralow trap densities (~ 10^12^ cm^−3^), and robust ambient redox stability. Integrated into planar NIR photodetectors, they deliver a responsivity of 0.51 A W^−1^ at 900 nm, a specific detectivity of 3.6 × 10^12^ Jones, rapid switching (~ 188 μs), and long-term operational durability over 25,000 on/off cycles. These findings establish coordination-guided crystallization as a platform concept for redox-stable, low-temperature integration of Sn–Pb SCTFs, opening pathways toward scalable, high-performance NIR optoelectronics.

## Experimental Section

### Materials

All chemicals were obtained from commercial sources and used without further purification. Organic halides (MAI and FAI, 99.99% purity) were from Great Cell Solar Materials. Thermo Fishers supplied PbI_2_ and SnI_2_ (99.999%). Cesium iodide (CsI, 99.9%), SnF_2_ (99.999%), PTAA (poly (triaryl amine)), dimethylformamide (DMF, 99.8% anhydrous), dimethyl sulfoxide (DMSO, 99.9% anhydrous), propylene carbonate (PC), tetramethylene sulfone (TMS), acetonitrile (ACN), and ethyl acetate (99.8% anhydrous) were from Sigma-Aldrich. MeO-2PACz (> 98%) was from Tokyo Chemical Industry.

### Preparation of Substrates and Deposition of Hole Transport Layers

Indium tin oxide (ITO) glasses (5 cm × 5 cm) were cleaned with soap, deionized water, and anhydrous ethanol, then treated with ultraviolet ozone (UVO) for 20 min. [2-(3,6-dimethoxy-9H-carbazol-9-yl)ethyl] phosphonic acid (MeO-2PACz) solution (1 mg mL^−1^ in ethanol) was spin-coated onto the substrates for 30 s at 3000 rpm, followed by annealing at 100 °C for 10 min. For comparison, poly(triarylamine) (PTAA) solution (1.5 mg mL^−1^ in chlorobenzene) was spin-coated at 4000 rpm for 30 s, then also annealed at 100 °C for 10 min. All procedures, except substrate cleaning and Sn–Pb perovskite single-crystal growth, including solution preparation, and thin single-crystal growth were conducted in an Ar-filled environment to prevent moisture-related issues.

#### Synthesis of Perovskite Bulk and Thin Single Crystals

Tin–lead mixed perovskite single crystals with varying thickness were synthesized using retrograde crystallization for thick Sn–Pb perovskite single crystals and space-confined retrograde crystallization for thin Sn–Pb perovskite single-crystal films. A 1.2 M saturated solution was prepared with a composition of FA_0.55_MA_0.4_Cs_0.05_Sn_0.5_Pb_0.5_I_3_, mixed in γ-butyrolactone (GBL) with cosolvents (ACN, TMS, and PC) at optimal volume ratios, stirred overnight at room temperature. The molar ratios for methylammonium iodide (MAI) and formamidine iodide (FAI) were 0.4:0.6, and for tin iodide (SnI_2_) and lead iodide (PbI_2_), they were 0.5:0.5. Cesium iodide (CsI) was added in excess (0.5 mol%), with SnF_2_ (10 mol% relative to SnI_2_). Tin powders (5 mg mL^−1^) were included to reduce Sn^4+^ in the precursor solution. The final solution was filtered through a 0.22 μm polytetrafluoroethylene (PTFE) membrane to remove any remaining tin powders before crystal growth. For bulk crystals, the filtered solution was transferred to a preheated oil bath under open atmospheric conditions to grow seed crystals. The temperature was gradually increased from room temperature to 45 or 100 °C, depending on the solvent matrix, allowing precise control of the crystal growth rate. For thin single crystals, optimal precursor amount was placed on a MeO-2PACz-coated (or PTAA-coated) substrate preheated to the solution temperature. Another similarly coated substrate was placed on top, creating a confined space for crystal growth. The temperature was gradually increased to induce nucleation, first at a rate of 3 °C up to 45 °C, then to 60 °C at 2 °C for thicker crystals. Once crystallization was complete, the substrates were separated with a razor blade and cooled to room temperature on a hotplate. Crystal sizes ranged from 1 to 2 mm^2^. Then, C_60_ (20 nm) and 5 nm of bathocuproine (BCP) were thermally evaporated at 0.1 Å s^−1^ to form the electron transport layer. The edges of the single crystals were then masked with Kapton tape to prevent short-circuiting between the top electrode (Cu) and ITO. Finally, Cu (80 nm) was evaporated at 1 Å s^−1^ to complete the device. Each device was photo masked with an area of 0.0049 cm^2^ before photovoltaic testing.

#### Solubility Test

The as-grown FA_0.55_MA_0.4_Cs_0.05_Sn_0.5_Pb_0.5_I_3_ crystals were crushed into powder and gradually added to 1 ml of GBL or GBL/Cosolvents mixture at the desired temperature. This process stopped when saturation was reached.

### Characterization of Devices and Films

Powder X-ray diffraction (XRD) analysis was performed using an X'Pert instrument from PANalytical with Cu Kα beams. UV–Vis–NIR spectroscopy (Cary 5000, Agilent Technologies) was used to measure the absorption spectra. Field emission scanning electron microscopy (FE-SEM, JSM-6701F, JEOL) was used to study surface morphology and obtain cross-sectional images. Energy-dispersive X-ray spectroscopy (EDS) was performed using a HITACHI S-4800 equipped with a Horiba EX-250. A confocal scanning microscope (MicroTime-200, PicoQuant, Germany) was used for time-resolved photoluminescence (TRPL) measurements. Pulsed laser excitation (470 nm with a pulse width of ~ 30 ps and an average power of ~ 1 μW at a repetition rate of 10 MHz) and a time-correlated single-photon counting system were utilized to analyze the emission. Steady-state photoluminescence (PL) spectra of the crystals were examined using two PL systems: One was with a monochromator (SP-2150i, Acton) equipped with an NIR detector (InGaAs, Acton ID-441) and a picosecond-pulsed laser as the excitation source. A pulsed diode-laser head (LDH-P–C-405, PicoQuant) coupled with a laser diode driver (PDL 800-B, PicoQuant) was used as the excitation source at a wavelength of 400 nm with a repetition rate of 80 MHz. Another was LabRAM Evolution, HORIBA, equipped with an NIR detector (InGaAs) and a 785-nm continuous-wave (CW) laser as the excitation source. HRTEM and SAED images were carried out on a JEOL ARM-200F electron microscope, operating at 200 kV. The information of element distribution of the single crystals was collected with EDS. X-ray photoelectron spectroscopy (XPS) was performed using a Theta Probe AR-XPS system (Thermo Fisher Scientific) with a monochromatic Al Kα X-ray source. Binding energies were provided with respect to the C 1* s* peak of the hydrocarbons at 285.0 eV. The spectra were analyzed using CASA XPS software. The devices were tested using a Newport solar simulator (Oriel Solar 3A Class, 94023A) equipped with a xenon lamp calibrated to AM 1.5G (100 mW cm^−2^). J–V characteristics of the solar devices were measured using a Keithley 2400 source meter. The measurements were conducted in both forward and reverse directions at a scan rate of 100 mV s^−1^ with a voltage step of 20 mV. No preconditioning such as light soaking was applied. Space-charge-limited current (SCLC) measurements were performed on ITO/perovskite single crystals/Cu structures using a Keithley 2400 source meter. The measurement range was from 0.01 to 2.5 V in the dark. Dark I*–*V measurement involved sweeping the voltage continuously from −1.2 to 1.2 V with a step of 0.01 V. Optical images were taken by optical microscope (Olympus DX51). The dark I–V characteristics for the SCLC analysis were carried out via a mechanical probe station and source meter (Keithley 4200-SCS, Keithley) under dark conditions. For the photodetector properties, J–V curves were measured using a source meter (Keithley 4200-SCS) by sweeping the voltage from 1 to −1 V under dark and under light using an 830-nm laser with various light intensities. The active areas of the devices were 0.0049 cm^2^. The EQE, spectral responsivity, and spectral detectivity were measured using a tunable light source for QE (TLS-300XU, Newport). The time-dependent on/off modulations were measured using a chopper to uniformly turn on/off the light source with a light frequency of 10 Hz (for cycle-dependent modulation) under zero bias.

## Results and Discussion

### Cosolvent Engineering for Low-Temperature Sn–Pb Single-Crystal Growth

**Fig. 1:**
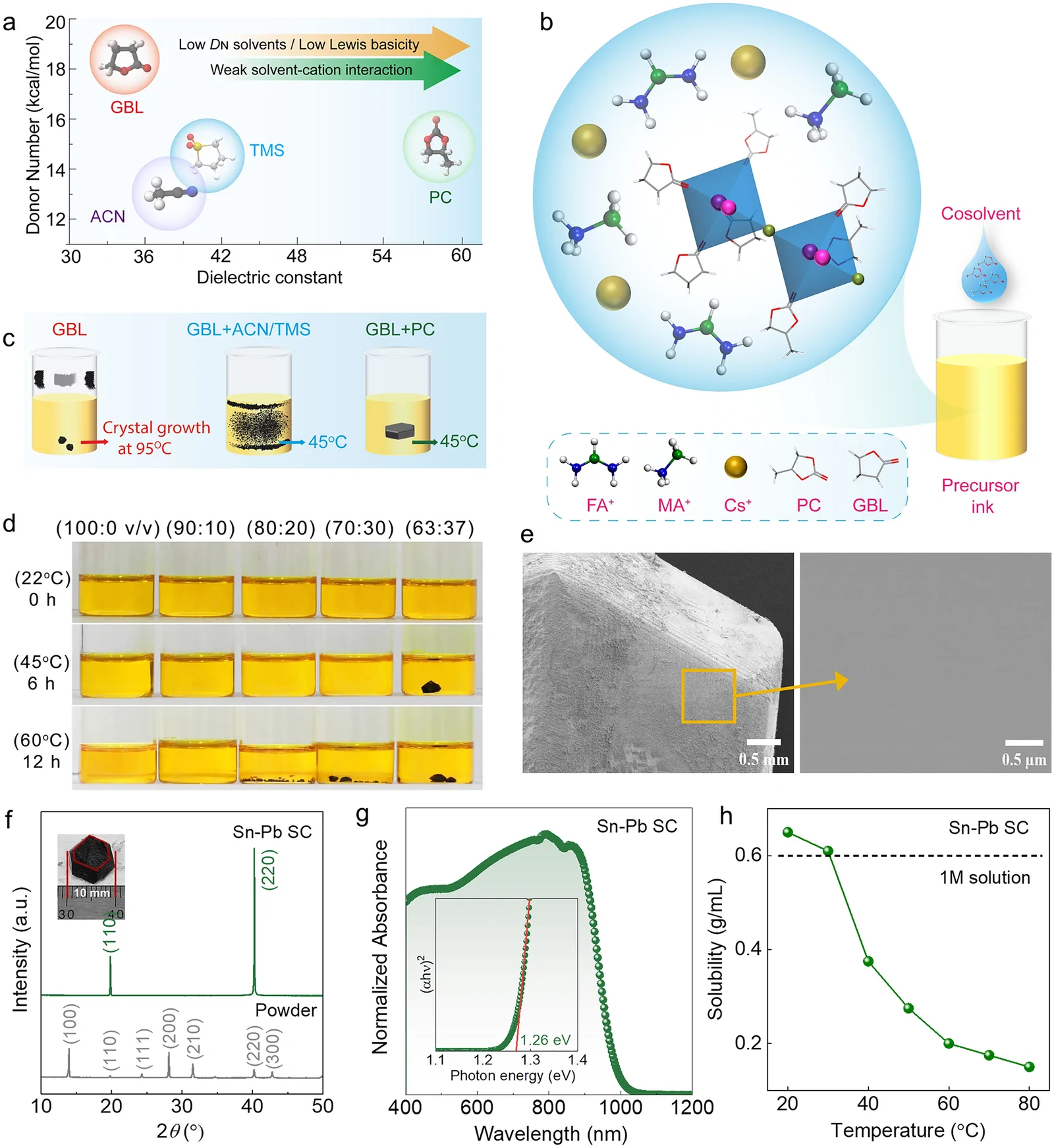
Coordination-modulated crystallization strategy and structural characterization of Sn–Pb single crystals. **a** Donor numbers and dielectric constants of selected cosolvents (see Table S1). **b** Schematic of the cosolvent-assisted inverse-temperature crystallization strategy. **c** Crystal growth onset temperatures in different solvent systems**. d** Digital photographs of precursor solutions with varying GBL:PC volume ratios before and after crystallization. **e** SEM image of a grain-boundary-free crystal surface. **f** XRD patterns with high-resolution scan of the (110) reflection (inset: optical image of a centimeter-scale crystal). **g** UV–Vis–NIR spectrum showing a sharp edge corresponding to a bandgap of ~ 1.26 eV. **h** Solubility trends of Sn–Pb precursors in GBL/PC systems as a function of volume ratio

To identify coordination environments suitable for low-temperature growth of Sn–Pb perovskite single crystals, we systematically screened solvents spanning a wide range of Gutmann donor numbers to evaluate their influence on precursor stability and nucleation behavior. The Gutmann Donor Number (*D*_N_), a quantitative measure of solvent Lewis’s basicity, served as a framework to assess solvent–metal coordination strength [27]. High-*D*_N_ solvents commonly used in perovskite processing—dimethyl sulfoxide (DMSO; *D*_N_ = 26.0 kcal mol^−1^) and N, N-dimethylformamide (DMF; *D*_N_ = 22.4 kcal mol^−1^)—formed strong [M–solvent]^2+^ adducts with Sn^2+^ and Pb^2+^, which competitively inhibited halide complexation and fully suppressed crystallization under ITC growth conditions [28]. Conversely, weakly coordinating solvents such as acetonitrile (ACN; *D*_N_ = 14.1 kcal mol^−1^) and tetramethylene sulfone (TMS; *D*_N_ = 14.7 kcal mol^−1^) lacked sufficient coordination strength to stabilize precursor complexes, resulting in rapid precipitation at room temperature (Figs. S1 and S2). Together, these findings define a coordination window—where strong coordination suppresses halide binding and weak coordination leads to premature precipitation—within which stable metal–halide complexes are likely to form and support controlled crystallization. This framework is illustrated by plotting the selected solvents on a *D*_N_–dielectric constant map (Fig. 1a), alongside a solvation model highlighting how low-*D*_N_ cosolvents promote halide complexation via weakened metal–solvent interactions (Fig. 1b). Mechanistically, the low-*D*_N_ cosolvent strategy attenuates metal–solvent coordination, strengthens metal–iodide complexation, and lowers the nucleation energy barrier, thereby promoting gradual, spatially uniform crystal growth under ITC conditions. To experimentally probe this coordination window, we selected γ-butyrolactone (GBL; *D*_N_ = 17.8 kcal mol^−1^) as a primary solvent due to its intermediate donor strength, which stabilized Sn–Pb precursors without triggering premature crystallization [27, 29].

The perovskite composition FA_0.55_MA_0.4_Cs_0.05_Sn_0.5_Pb_0.5_I_3_ was formulated by dissolving equimolar FAI, MAI, CsI, SnI_2_, and PbI_2_ in GBL at a concentration of 1.2 M. Precursor solutions were sealed in closed vials and subjected to ITC growth via slow thermal ramping in an oil bath (see Methods). While GBL-based solutions supported retrograde solubility, nucleation occurred only after prolonged heating at ~ 85 °C and typically yielded dendritic morphologies due to uncontrolled supersaturation (Fig. 1c). To modulate coordination strength and reduce the nucleation barrier, we introduced propylene carbonate (PC; *D*_N_ = 15.1 kcal mol^−1^) as a cosolvent to attenuate metal–solvent interactions while preserving retrograde solubility. GBL:PC mixtures with volume ratios from 100:0 to 63:37 were systematically evaluated under identical thermal conditions. FTIR of precursor inks shows a ν(C = O) red-shift in GBL upon SnI_2_/PbI_2_ addition, whereas no resolvable shift is observed in GBL/PC (63:37), indicating weaker direct carbonyl coordination. A neat PC spectrum (carbonate ν(C = O) ~ 1790 cm^−1^) is provided for reference. Together with the higher dielectric constant of the mixture, this supports donor number- and dielectric-driven moderation of coordination while preserving solubility (Fig. S3; Table S1). As PC content increased, the nucleation onset temperature decreased—from ~ 85 °C in pure GBL to ~ 60 °C at 80:20, and further to ~ 36 °C in the 63:37 mixture—while crystal morphology evolved from dendritic to sharply faceted. The 63:37 formulation consistently enabled millimeter-scale crystal growth at 45 °C within 6 h, without visible discoloration, indicating redox resilience at prolonged growth period (Fig. 1d). GBL provides moderate Lewis coordination and inverse-temperature solubility, while PC contributes high dielectric screening with weak coordination; the GBL/PC (63:37) mixture, therefore, balances binding strength and ionic dissociation to enable controlled nucleation and growth (Figs. 1b and S3; Table S1). The resulting crystals exhibited well-defined facets, uniform thickness, and lateral dimensions exceeding 1 mm, as confirmed by scanning electron microscopy (SEM) (Fig. 1e). EDS elemental maps of the as-grown crystals show spatially even Sn, Pb, and I distributions across the mapped area (Fig. S4). X-ray diffraction (XRD) patterns showed intense, narrow peaks indexed to the cubic perovskite phase with no detectable secondary phases (Fig. 1f), while single-crystal XRD confirmed a preferred (110)/(220) orientation, indicative of high crystallographic order [30]. A standard reference diffraction pattern for mixed Sn–Pb perovskite single crystals is not available in major crystallographic databases. The dominant (110)/(220) reflections are consistent with oriented nucleation and preferential stabilization of the (110) family during inverse-temperature crystallization (Figs. 1f and S12) [31–33]. UV–Vis–NIR spectra exhibited a sharp onset at ~ 988 nm, corresponding to a direct bandgap of ~ 1.26 eV (Fig. 1g), well aligned with near-infrared detection requirements [26]. The shallow visible-range undulations in the absorbance originate from optical interference within the parallel-faced, micrometer- to millimeter-thick single crystals, consistent with prior reports [34–36]. Figure S5 reports the raw absorbance of the Sn–Pb single crystal used in Fig. 1g and the corresponding extinction coefficient *k(*λ) corroborating the strong band edge at ~ 988 nm and strong NIR response. Solubility analysis confirmed a retrograde profile in the GBL:PC system (Fig. 1h), validating the thermodynamic conditions necessary for controlled supersaturation and directional crystal growth under ITC.

### Confined Growth of Planar Sn–Pb Single-Crystal Thin Films

**Fig. 2:**
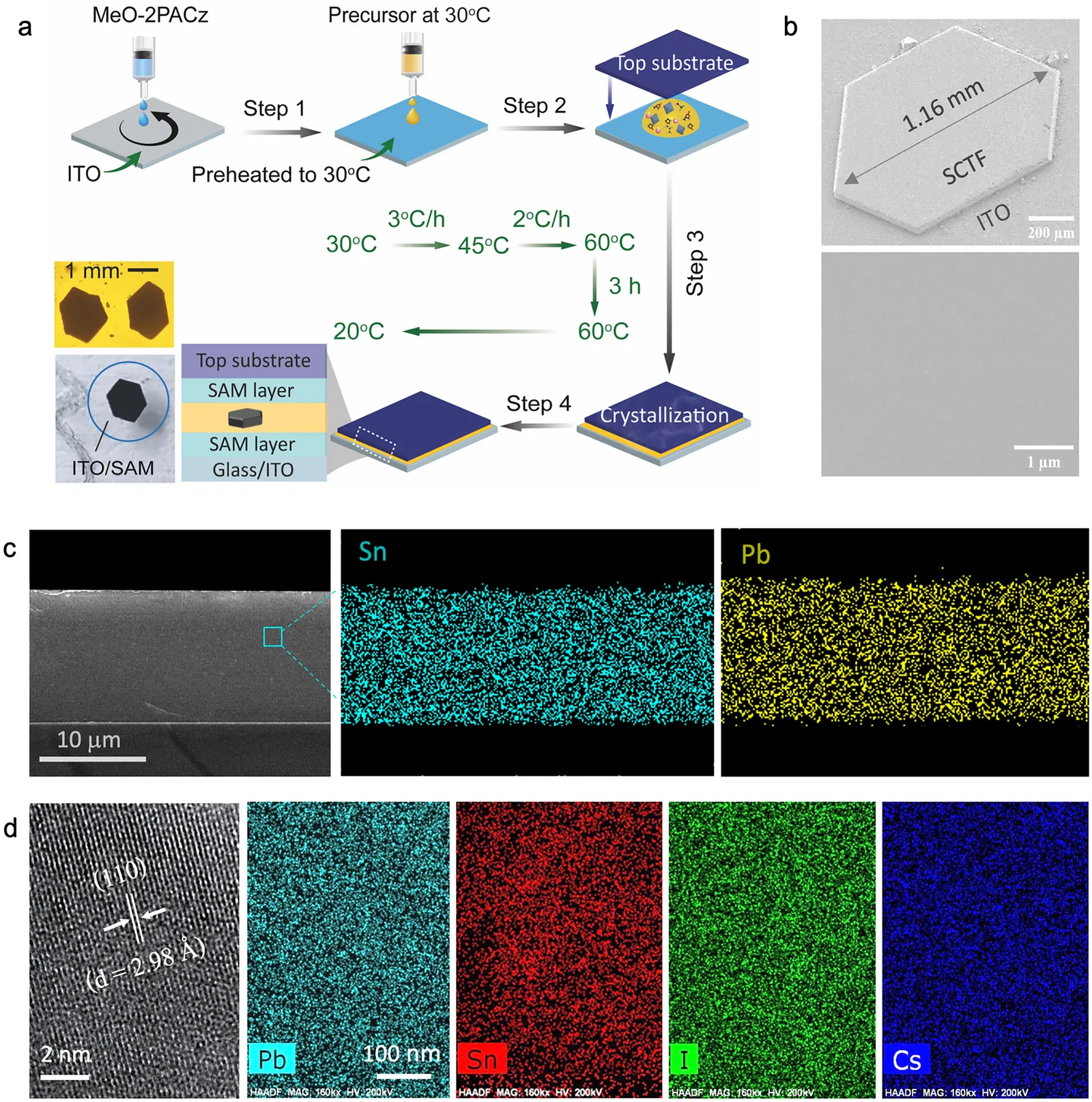
Growth and structural characterization of Sn–Pb single-crystal thin films. **a** Schematic of the spatially confined inverse-temperature crystallization strategy. **b** Top-view SEM images of a representative SCTF at low and high magnifications (scale bars: 200 μm and 1 μm). **c** Cross-sectional EDS mapping of a 12 μm-thick SCTF showing uniform elemental distribution of Sn, Pb, and I. **d** High-resolution TEM image of the (110) lattice plane and corresponding elemental maps of Pb, Sn, I, and Cs (scale bar: 100 nm)

Having established the coordination window for low-temperature growth of bulk Sn–Pb single crystals, we next aimed to translate these insights into a planar, substrate-integrated geometry compatible with monolithic optoelectronic devices. Monolithic integration denotes direct, transfer-free growth of micrometer-thick Sn–Pb SCTFs on conducting substrates by spatially confined crystallization. To this end, we adapted the ITC method into a spatially confined growth configuration that promotes lateral growth of single-crystal thin films (SCTFs) directly on conductive substrates. In this geometry, precursor solutions prepared using the optimized GBL/PC (63:37 v/v) cosolvent system were enclosed between two parallel substrates, facilitating directional extension and sharply defined edges (Fig. 2a).

A two-step thermal ramp—from 30 to 45 °C at 3 °C h^−1^, followed by 45 to 60 °C at 2 °C h^−1^—was employed to promote uniform nucleation and maintain gradual supersaturation while minimizing thermal gradients and interfacial stress. This controlled heating protocol enabled reproducible SCTF growth with tunable thicknesses ranging from ~ 12 μm (3 h) to ~ 27 μm (18 h), without the need for post-thinning—highlighting the compatibility of this approach with direct device integration (Fig. S6). Single-crystal film thickness scales linearly with growth time at ~ 1.4 µm h^−1^ under fixed concentration (1.2 M) and gap (~ 100 µm), while lateral size increases from ~ 0.6 to ~ 1.4 mm between 3 and 12 h (Figs. S6 and S7; Tables S2 and S3).

To evaluate the role of interfacial chemistry, we systematically compared crystallization outcomes on ITO substrates coated with either the conventional hole-transporting polymer PTAA or a self-assembled monolayer (SAM) of MeO-2PACz. While PTAA resulted in poor surface coverage and frequent void formation (Fig. S8), MeO-2PACz improved wettability and interfacial compatibility, enabling uniform lateral nucleation and void-free crystal extension. The spatially confined crystallization process yielded SCTFs with excellent structural and compositional uniformity across device-relevant areas. SEM top-view images (Fig. 2b) revealed well-faceted hexagonal domains exceeding 1 mm in lateral dimension, with sharply defined edges and smooth surfaces, confirming directional, void-free lateral growth. Atomic force microscopy (AFM) measurements (Fig S9) further confirm that the films retain smooth, uniform surfaces (RMS roughness < 2 nm) across different growth durations, consistent with the high structural quality of the SCTFs. Cross-sectional SEM analysis (Fig. 2c) showed continuous film geometries with well-defined interfaces, while EDS elemental mapping (Figs. 2c and S10) confirmed homogeneous Sn and Pb distributions across the SCTF thickness—indicating successful metal incorporation and compositional homogeneity. At the nanoscale, high-resolution transmission electron microscopy (HRTEM) imaging (Fig. 2d) resolved lattice fringes with a spacing of 2.98 Å, corresponding to the (110) planes of the cubic perovskite phase, confirming high crystallographic order [37]. Complementary scanning transmission electron microscopy (STEM)–EDS elemental maps (Figs. 2d and S11) showed uniform distributions of Pb, Sn, I, and Cs, further validating compositional homogeneity throughout the multiplication lattice. The absence of detectable ion clustering or phase inhomogeneity highlights the effectiveness of coordination-modulated growth in preserving redox stability and stoichiometric fidelity. Compositional analyses (SEM–EDS, STEM-EDS, and XPS) confirm near-equimolar B-site incorporation with Sn:(Sn + Pb) ≈ 0.50 and I:(Sn + Pb) ≈ 2.9, with Cs ≈ 0.05 (Figs. 2c, d and S10–S11; Table S5). Because FA/MA is not directly resolved by EDS/XPS, a Vegard’s law consistency check (Eqs. S1 and S2) using the measured lattice constant (a = 6.291 Å) and Cs ≈ 0.05 yields FA:MA ≈ 0.58:0.37, in close agreement with the nominal FA_0.55_MA_0.40_. Together, these results establish the cosolvent-guided spatially confined ITC platform as a robust and scalable approach for growing redox-stable, micrometer-thick Sn–Pb perovskite SCTFs with high crystallographic order and compositional uniformity.

### Optoelectronic Quality and Redox Stability of Sn–Pb SCTFs

**Fig. 3:**
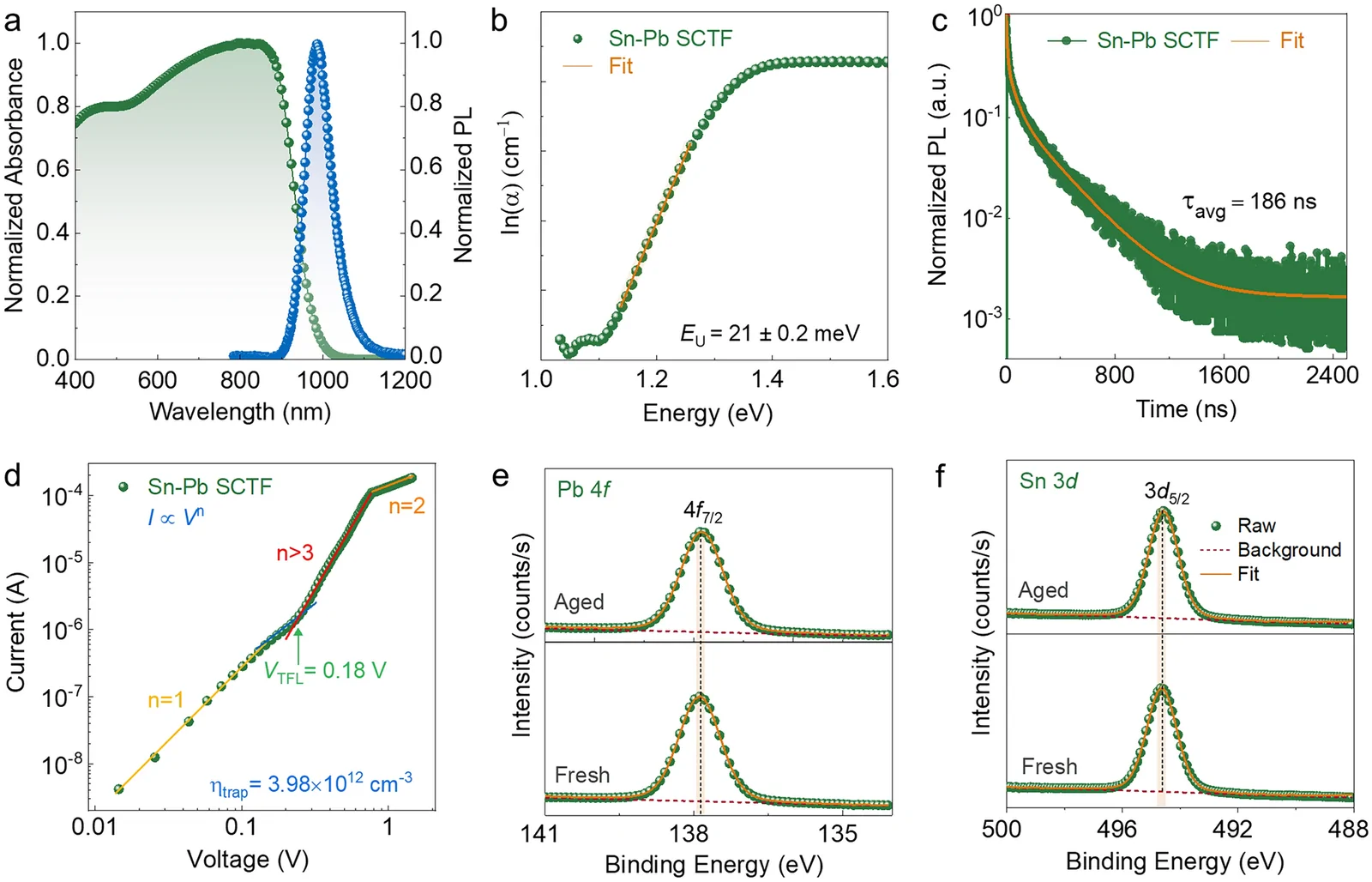
Optoelectronic and surface chemical properties of Sn–Pb single-crystal thin films. **a** UV–Vis–NIR and photoluminescence spectra showing a sharp absorption onset (~ 1.26 eV) and strong band-edge emission. **b** Urbach energy extracted from the absorption tail, indicating low energetic disorder. **c** Time-resolved photoluminescence decay fitted with a tri-exponential model. **d** SCLC measurements for estimating trap-state density. **e, f** XPS spectra of Pb 4*f* and Sn 3*d* core levels for fresh and humidity-aged films (100 h at 20% RH)

To evaluate the structural and optoelectronic quality of Sn–Pb SCTFs for NIR optoelectronic applications, we conducted comprehensive crystallographic and optoelectronic characterizations. Out-of-plane XRD 2θ scans (Fig. S12) revealed intense reflections at 19.82° and 40.26°, corresponding to the (110) and (220) planes of the cubic perovskite lattice. The narrow full-width at half-maximum (FWHM) values—0.025° and 0.028°, respectively—indicate high crystallographic order and minimal structural disorder, consistent with a well-faceted single-crystalline framework*.* UV–Vis–NIR spectra (Fig. 3a) exhibited a sharp onset at ~ 988 nm, consistent with a direct bandgap of ~ 1.26 eV, as confirmed by both Tauc fitting and steady-state photoluminescence (PL) [38]. The sub-bandgap region, analyzed using the Urbach model (Fig. 3b), yielded an Urbach energy (Eᵤ) of 21 ± 0.2 meV, reflecting low energetic disorder and minimal tail-state formation. Time-resolved PL (TRPL) measurements on ITO/MeO-2PACz/Sn–Pb SCTF stacks (Fig. 3c) revealed tri-exponential decay dynamics with a weighted average lifetime of 186 ns (τ_1_​ = 7.73 ns, τ_2_ = 54.72 ns, and τ_3_ = 277.17 ns; Table S4), consistent with suppressed non-radiative channels and long-range carrier diffusion*.*

To directly quantify trap-state density, we fabricated hole-only ITO/MeO-2PACz/Sn–Pb SCTF/Cu devices and performed space-charge-limited current (SCLC) measurements under dark conditions (Fig. 3d) [39]. The current–voltage response shows a transition from Ohmic to trap-filled-limited (TFL) regimes at *V*_TFL_ ≈ 0.18 V, from which we estimated (Eq. S3) a trap density of 3.98 × 10^12^ cm^−3^—3–4 orders of magnitude lower than typical polycrystalline Sn–Pb perovskites [40–43]*.* The ultralow trap density reflects coordination-modulated growth that (i) yields grain-boundary-free films, (ii) stabilizes Sn^2+^ against oxidation during low-temperature crystallization in the GBL/PC matrix, and (iii) promotes confined step-flow that preserves lattice coherence and stoichiometric uniformity. To assess redox resilience, we tracked the surface chemical states via X-ray photoelectron spectroscopy (XPS) after 100 h exposure to 20% relative humidity. Core-level spectra of Pb 4*f* and Sn 3*d* (Fig. 3e, f) retained their spin–orbit splitting energies (4.9 and 8.4 eV, respectively), with no detectable signals of Sn^4+^ or metallic Pb^0^—indicating the absence of surface oxidation or reduction pathways [38]*.* This intrinsic redox stability—achieved without encapsulation or passivation—reflects the combined effects of a grain-boundary-free morphology and a stabilized coordination environment established during low-temperature crystal growth. Additional I 3*d*, survey spectra and elemental ratios are presented in Fig. S13 and Table S5. Collectively, these results establish Sn–Pb SCTFs as a high-quality optoelectronic platform, combining sharp band-edge absorption, extended carrier lifetimes, ultralow trap densities, and excellent environmental stability. Notably, these performance metrics were achieved without post-deposition surface passivation, underscoring the critical role of coordination-guided crystallization in suppressing intrinsic and extrinsic defects*.* Relative to prior Sn- and Sn–Pb single crystals cataloged by composition, growth route/temperature, thickness, and trap-state density (Table S6), and to chemically passivated polycrystalline films (Table S7), our SCTFs exhibit lower trap-state densities, improved redox stability, and straightforward planar integration.

### Device Integration and High-Performance NIR Photodetection

**Fig. 4:**
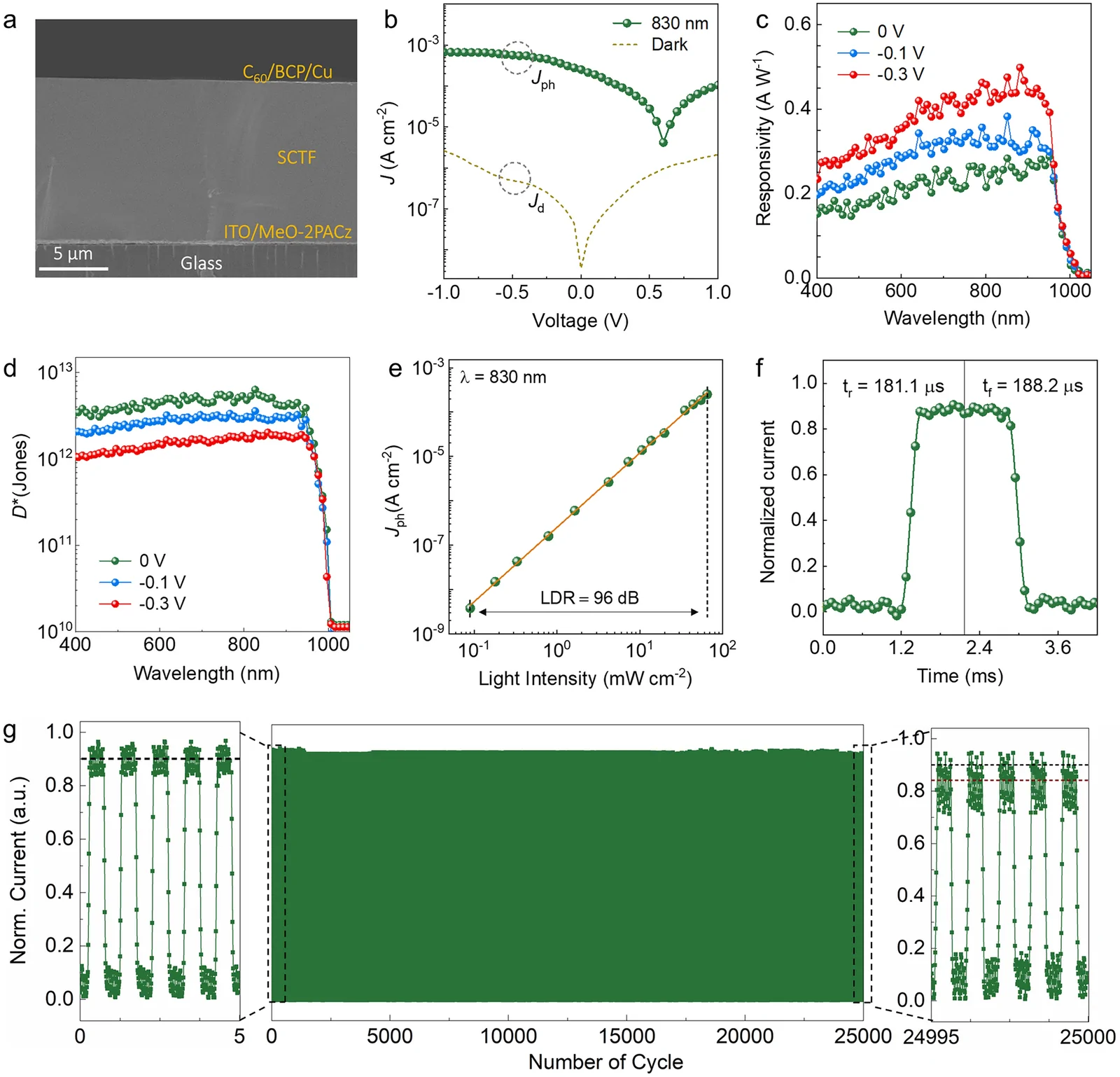
Photodetection performance of Sn–Pb single-crystal thin film devices. **a** Cross-sectional SEM image of the complete device architecture. **b** J–V characteristics in the dark and under 830 nm illumination (65.1 mW cm^−2^), showing low dark current and high on/off ratio. **c, d** Spectral responsivity and specific detectivity (0.0049 cm^2^) of single-crystal photodetector under different biases. **e** LDR measured at 0 V, following a power–law fit with slope β = 0.92.** f** Transient photoresponse under pulsed 830 nm illumination (10 Hz), revealing fast rise and decay times. **g** Operational stability measured under ambient air (22–25 °C, ~ 55% RH, unencapsulated) with 830 nm illumination (65.1 mW cm⁻^2^, 10 Hz modulation) over 25,000 continuous on/off cycles; insets show initial and final pulse segments

To translate the intrinsic optoelectronic quality of Sn–Pb SCTFs into device-level functionality, we fabricated planar NIR photodetectors based on an ITO/MeO-2PACz/Sn–Pb SCTF/C_60_/BCP/Cu architecture (Fig. 4a) [44]. J–V characteristics were recorded in ambient air over –1.0 to +1.0 V under 830 nm illumination at 65.1 mW cm^−2^ (and in the dark). The device shows a dark current density of ~ 3.5 × 10^–9^ A cm^−2^ at 0 V and an on/off ratio > 7.2 × 10^4^ (Fig. S14). The short-circuit photocurrent density reached ~ 2.5 × 10^–4^ A cm^−2^, enabled by broadband NIR absorption and efficient charge extraction across the crystalline interface [45, 46].

Spectral response analysis revealed a sharp external quantum efficiency (EQE) onset at ~ 988 nm (Fig. S15), closely matching the optical bandgap of the Sn–Pb absorber (~ 1.26 eV) [47]. At 900 nm, EQE improved from 42.3% at 0 V to 73.9% at –0.3 V, accompanied by an increase in responsivity from 0.28 to 0.51 A W^−1^ (Fig. 4c), reflecting built-in field-assisted carrier extraction and reduced interfacial recombination [48]*.* The higher responsivity at − 0.3 V reflects field-assisted extraction that reduces interfacial losses relative to 0 V; bias-dependent EQE/R and thickness-invariant response (Fig. S16) support an interface-limited short-circuit regime rather than bulk-defect limitation [49, 50]. Noise measurements are floor-limited for our 0.0053 cm^2^ pixel in the sub-picoampere regime, so specific detectivity is reported as a shot-noise upper bound [51]. At 900 nm, the specific detectivity D* reaches 3.6 × 10^12^ Jones at 0 V and remains ≥ 10^12^ Jones at − 0.1 V, and − 0.3 V, maintaining this level across the NIR spectrum (Fig. 4d). Thickness dependence (Fig. S16) shows comparable EQE/responsivity at ~ 12 and ~ 27 µm, but higher dark current density in the thicker film lowers the shot-noise-limited detectivity, favoring ~ 12 µm for higher performance.

To evaluate linearity, we examined the photocurrent response across illumination intensities spanning 0.09–65.1 mW cm^−2^. The light-intensity dependence photocurrent response followed a power–law dependence (*J*_ph_ ∝ I^β) with β = 0.92 (Fig. S17), confirming minimal trap-mediated recombination and efficient carrier extraction [52]*.* The linear dynamic range extended to ~ 96 dB (Fig. 4e), supporting utility in both low-light and high-i.ntensity sensing applications [53]. Transient response measurements under modulated 830 nm illumination revealed sub-millisecond switching kinetics, with rise and fall times of 181.1 and 188.2 μs, respectively (Fig. 4f). Trap-free SCLC on hole-only 12 µm SCTFs yields a mobility of μ = 2.18 ± 0.06 cm^2^ V⁻^1^ s⁻^1^ (Eq. S4). The measured switching constants exceed the transit-time bound estimated from μ and film thickness and match the RC/interface time constants extracted from small-signal analysis (Eq. S5), indicating that the temporal response is limited by RC/interface effects rather than bulk transport. Together with the low trap density and minimal structural disorder, these data support rapid rise/fall times and efficient carrier extraction (Eq. S5). These fast-switching kinetics represent a significant advancement over typical Sn–Pb polycrystalline photodetectors, which often suffer from trap-limited response times [54]. Operational stability (Fig. 4g) was assessed by on/off cycling at 10 Hz in ambient air (22–25 °C, ~ 55% RH) on unencapsulated devices under 830 nm illumination (65.1 mW cm^−2^) with failure defined at 95% photocurrent retention after 25,000 cycles. Table S8 summarizes comparable stability benchmarks for polycrystalline Sn–Pb photodetectors. This resilience arises from grain-boundary-free morphology and the redox stability conferred by low-temperature, coordination-guided crystallization. Cosolvent-engineered, spatially confined ITC is not size-limited—dimensions scale with gap, solvent volume, and thermal uniformity, already yielding millimeter-scale crystals; scaling to larger apertures requires preserving low dark current and minimizing series resistance via contact selection and optimized electrode geometry. Taken together, the low dark current, high specific detectivity, broadband responsivity, ~ 200-µs response, wide linear range, and exceptional ambient durability—achieved without passivation or encapsulation—establish Sn–Pb SCTFs as a strong basis for scalable NIR photodetectors.

## Conclusions

We present a coordination-modulated crystallization strategy that enables low-temperature growth of redox-stable Sn–Pb SCTFs using a low Lewis basicity γ-butyrolactone/propylene carbonate (GBL/PC) cosolvent system. By systematically tuning metal–solvent coordination strength, we delineate a narrow processing window that promotes halide-rich precursor complexation while mitigating oxidative degradation of Sn^2+^ species. This molecular-level control enables the growth of thickness-tunable, grain-boundary-free SCTFs exhibiting ultralow trap-state densities (~ 10^12^ cm^−3^) and intrinsic ambient stability—without requiring post-growth surface passivation. When integrated into planar near-infrared photodetectors, these SCTFs yield high responsivity, shot-noise-limited detectivity, sub-millisecond switching kinetics, and wide linear dynamic range, alongside stable operation over > 25,000 light-switching cycles under ambient conditions. These combined metrics surpass existing benchmarks for solution-processed Sn–Pb optoelectronic devices, validating the effectiveness of our coordination-guided crystallization approach. Beyond the Sn–Pb system, this cosolvent-directed strategy provides a generalizable platform for scalable, defect-suppressed single-crystal growth across perovskite compositions. By bridging crystallization control, structural purity, and device-grade integration, this work charts a path toward intrinsically stable, high-performance materials for next-generation optoelectronic technologies.

## Supplementary Information

Below is the link to the electronic supplementary material.

Supplementary file1 (DOCX 20322 kb)
