# Supplementary material for: Monolithic Integration of Redox-Stable Sn–Pb Halide Perovskite Single-Crystalline Films for Durable Near-Infrared Photodetection
Source: Nanomicro Lett. 2026 Jan 12;18:141. doi: 10.1007/s40820-025-01991-y (PMC12791097; doi:10.1007/s40820-025-01991-y)
Supplement: Supplementary file 1 — Supplementary file1 (DOCX 20322 kb) [file 40820_2025_1991_MOESM1_ESM.docx]

Supporting Information for

**Monolithic Integration of Redox-Stable Sn–Pb Halide Perovskite Single-Crystalline Films for Durable Near-Infrared Photodetection**

Rajendra Kumar Gunasekaran^1, 2, 3^*^†^, Jihoon Nam^1†^, Myeong-geun Choi^1^, Won Chang Choi^1^, Sunwoo Kim^1^, Doyun Im^1^, Yeonghun Yun^4^, Yun Hwa Hong^5^, Sang Hyeok Ryou^6^, Hyungwoo Lee^6^, Kwang Heo^5^, Sangwook Lee^1^*

^1^ School of Materials Science and Engineering, Kyungpook National University (KNU), Daegu 41566, Republic of Korea

^2^ School of Physics, The University of Sydney, NSW 2006, Australia

^3^ The University of Sydney Nano Institute (Sydney Nano), The University of Sydney, NSW 2006, Australia

^4^ Department Perovskite Tandem Solar Cells, Helmholtz-Zentrum Berlin für Materialien und Energie GmbH, 12489 Berlin, Germany

^5^ Department of Nanotechnology and Advanced Materials Engineering, Hybrid Materials Research Center (HMC), Sejong University (SJU), Seoul 05006, Republic of Korea

^6^ Department of Physics, Department of Energy Systems Research, Ajou University, Suwon 16499, Republic of Korea

^†^Rajendra Kumar Gunasekaran and Jihoon Nam contributed equally to this work.

^*^Corresponding authors. E-mail: [rajendrakumargvm@gmail.com](mailto:rajendrakumargvm@gmail.com) (Rajendra Kumar Gunasekaran); [wook2@knu.ac.kr](mailto:wook2@knu.ac.kr) (Sangwook Lee)

# S1 Supplementary Figures and Tables


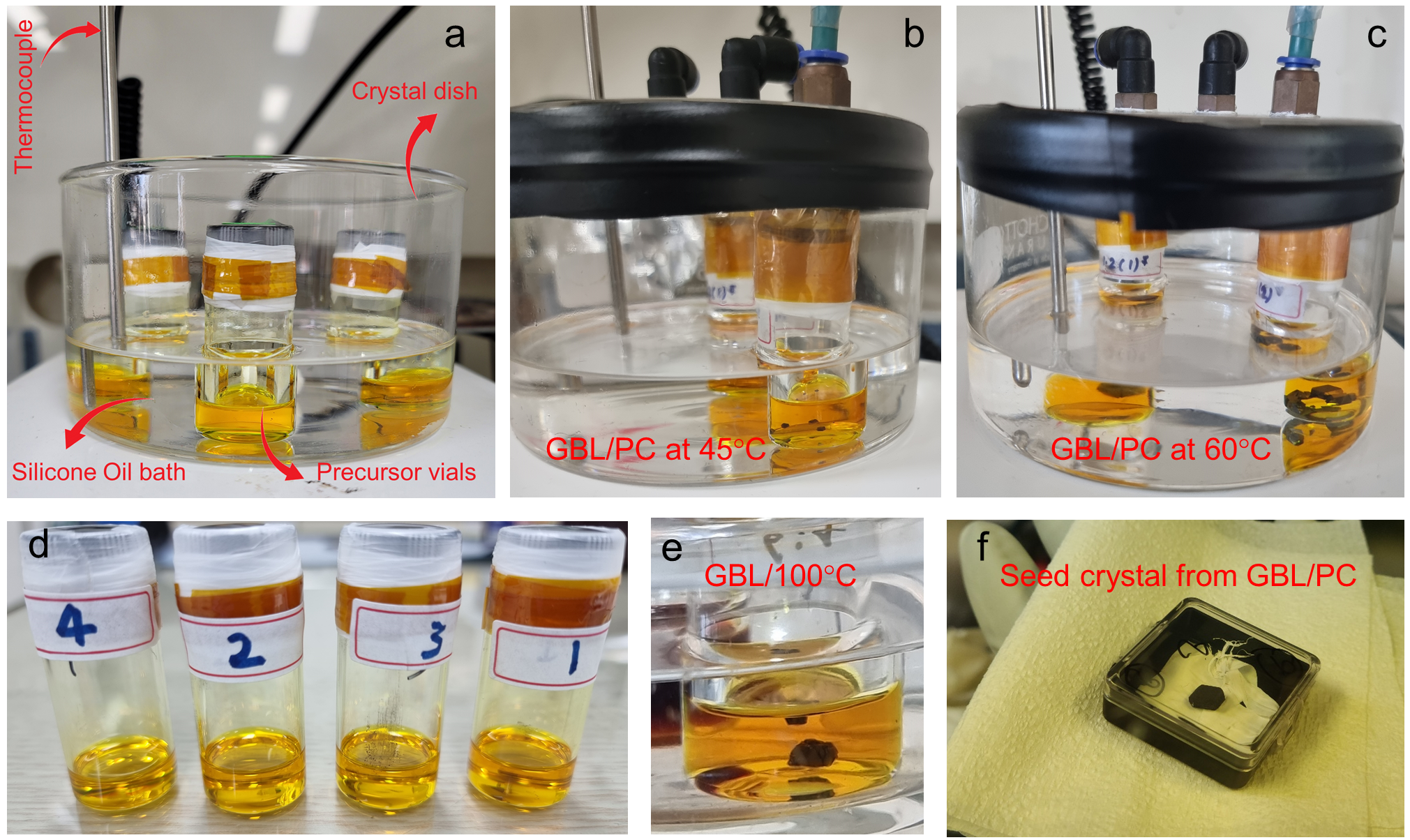


**Fig. S1** Photographs of the experimental setup and crystallization behavior during Sn-Pb single-crystal growth. (**a**) Initial setup for inverse temperature crystallization. (**b, c**) Crystal growth stages in GBL/PC (63:37 v/v) cosolvent system at 45 °C and 60 °C, respectively. (**d**) Precursor solutions prepared with different solvent mixtures: (1) GBL only, (2) GBL + PC, (3) GBL + ACN, and (4) GBL + TMS. (**e**) GBL-based precursor after heating at 100 °C for 12 h. (**f**) Seed crystals obtained from the GBL/PC solvent system

We first investigated the coordination effect of different solvents on mixed Sn–Pb perovskite single crystals (SCs) using the inverse temperature crystallization (ITC) method, wherein precursor solubility decreases with increasing temperature [S1]. Three commonly used solvents—DMF, DMSO, and GBL—were evaluated both individually and in combination. The experimental setup for bulk SC growth is shown in Fig. S1. Solutions prepared with DMF or DMSO remained clear even after extended heating at elevated temperatures, showing little to no precipitation and failing to initiate crystal nucleation. In contrast, GBL-based precursors showed visible precipitation above 95 °C, yielding seed crystals with irregular shapes and poor morphology. Further heating led to partial or complete re-dissolution of the crystals, indicating instability under prolonged thermal conditions. To improve control over solvent–precursor interactions, 2 vol.% formic acid was added to the GBL solution [S2], which induced nucleation and led to the formation of numerous small crystals upon heating at 65 °C for 12 h (Fig. S2). This result underscores the sensitivity of the crystallization process to solvent coordination strength and additive chemistry.

**
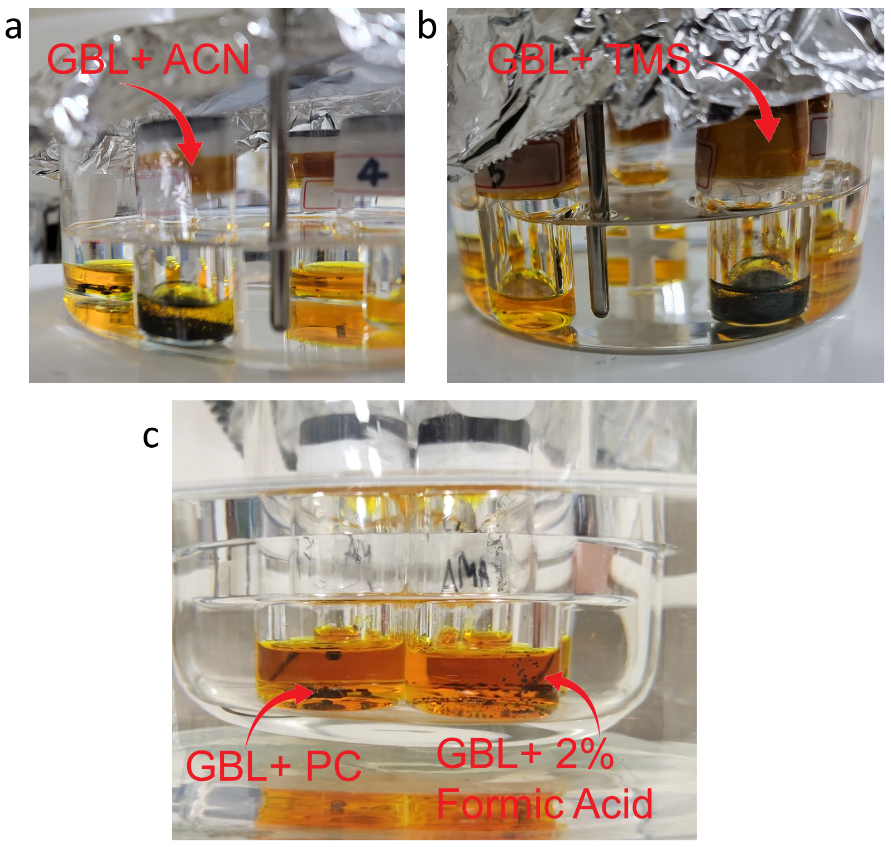
**

**Fig. S2** Photographs of halide Sn–Pb perovskite crystals formed in precursor solutions with various solvent combinations: **a** GBL + ACN, **b** GBL + TMS, **c** GBL + PC and GBL + 2% formic acid. Crystals in GBL + ACN and GBL + TMS systems precipitated rapidly at 45 °C, indicating uncontrolled nucleation. In contrast, GBL + PC enabled gradual crystal growth at 45 °C over 5 h, producing larger and more uniform crystals. The GBL + 2% formic acid mixture yielded irregular small crystals after 12 h at 65 °C


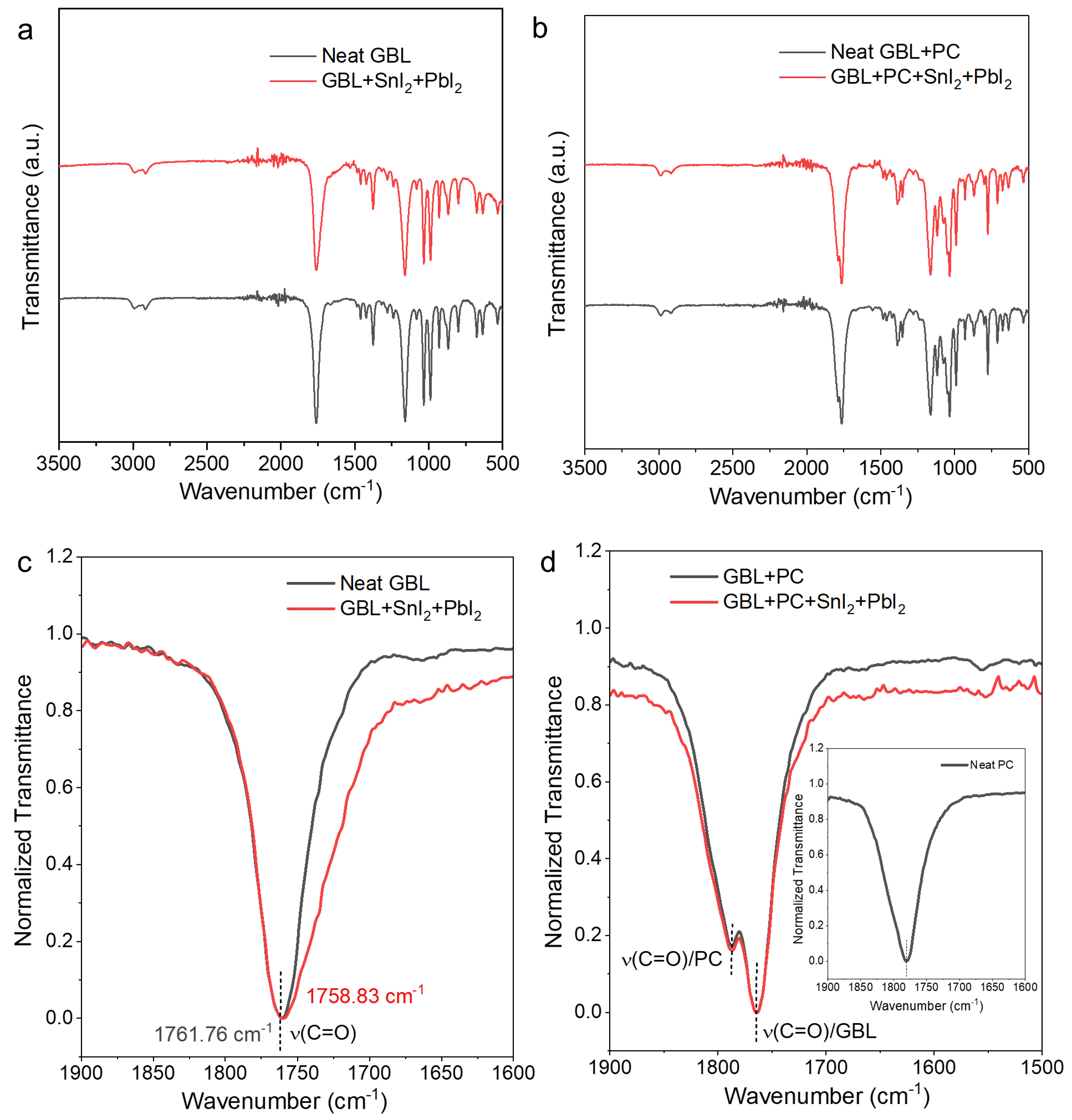


**Fig. S3** FTIR of solvent and precursor solutions. (**a**) Full-range FTIR spectra of neat GBL and GBL+SnI_2_+PbI_2_. (**b**) Full-range spectra of neat GBL+PC (63:37 v/v) and GBL+PC+SnI_2_+PbI_2_. (**c**) Carbonyl region (1900–1600 cm⁻^1^) of GBL with and without SnI_2_+PbI_2_. (**d**) Carbonyl region of GBL+PC mixtures with and without SnI_2_+PbI_2_. Inset: neat PC spectrum showing the carbonate ν(C=O) band (~1790 cm⁻^1^)


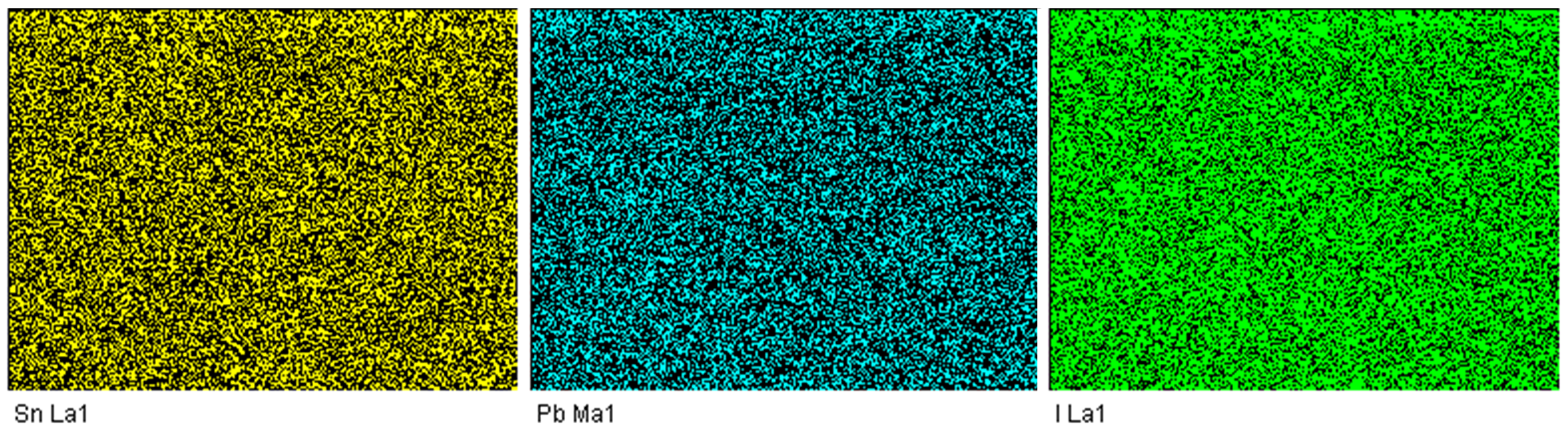


**
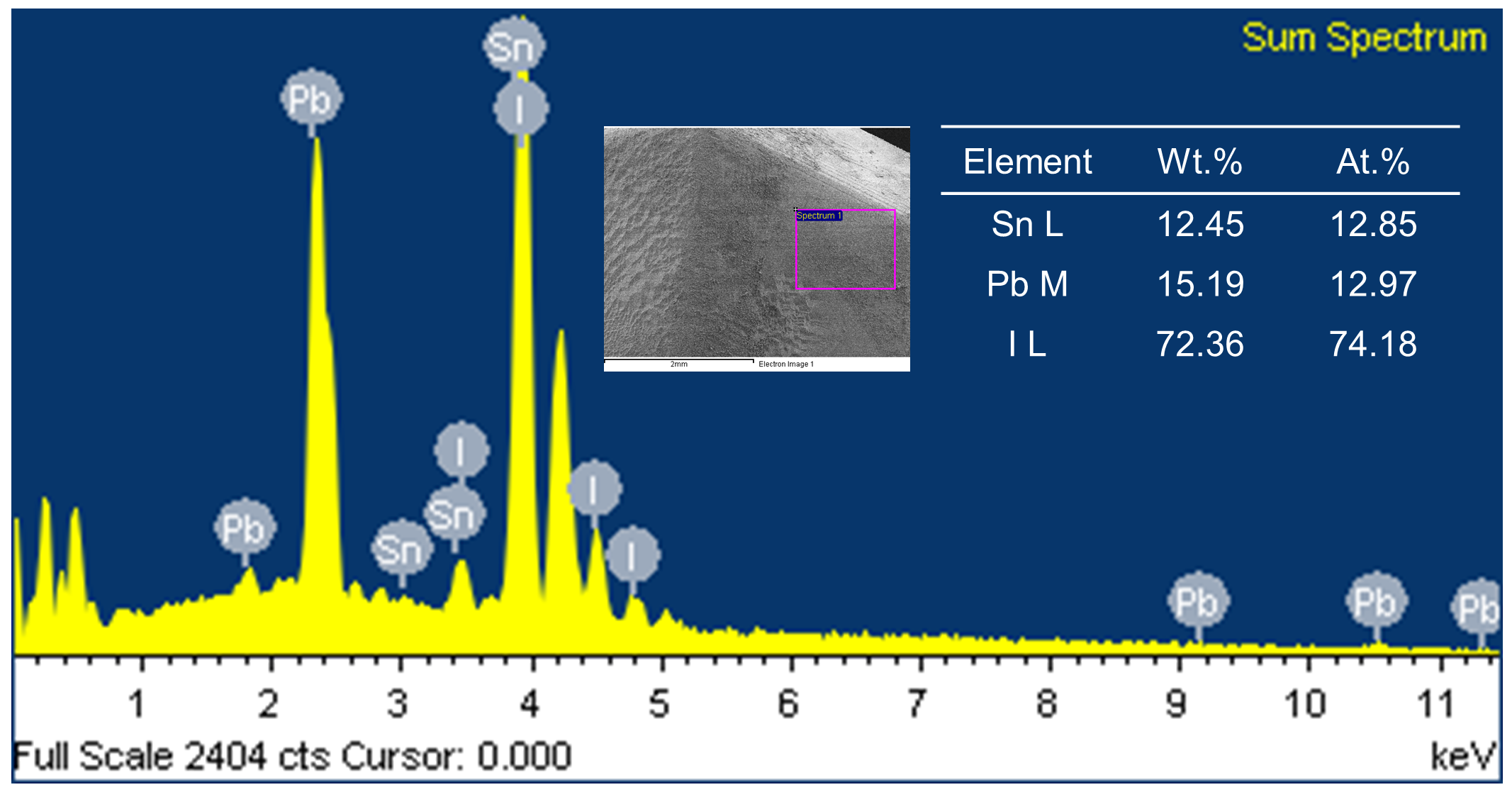
**

**Fig. S4** Energy-dispersive X-ray spectroscopy (EDS) elemental mapping of as-grown Sn–Pb single crystals, displaying spatially even signal distributions of tin (Sn), lead (Pb), and iodine (I) across the mapped area


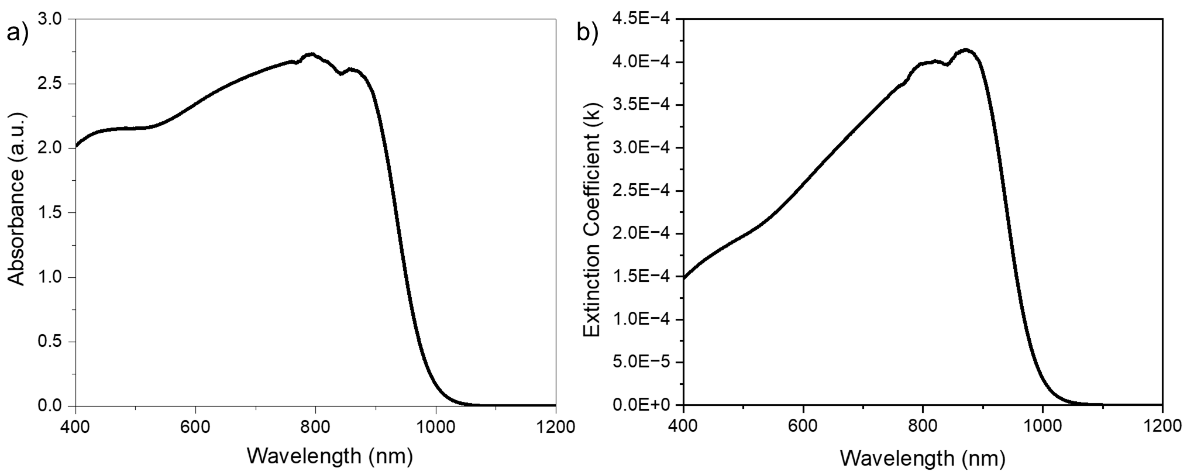


**Fig. S5** Optical spectra and derived extinction coefficients of the Sn–Pb single crystal. (**a**) UV–vis-NIR absorbance spectrum of the crystal used in Fig. 1g. (**b**) Extinction coefficient *k(*λ) derived from *k*(λ) = αλ/(4π); α was obtained from absorbance via α = 2.303×A/*d* (*d* =1.00 mm). The spectra reproduce the Fig. 1g trend and show a sharp absorption edge near ~988 nm (E_g_ = 1.26 eV)

**
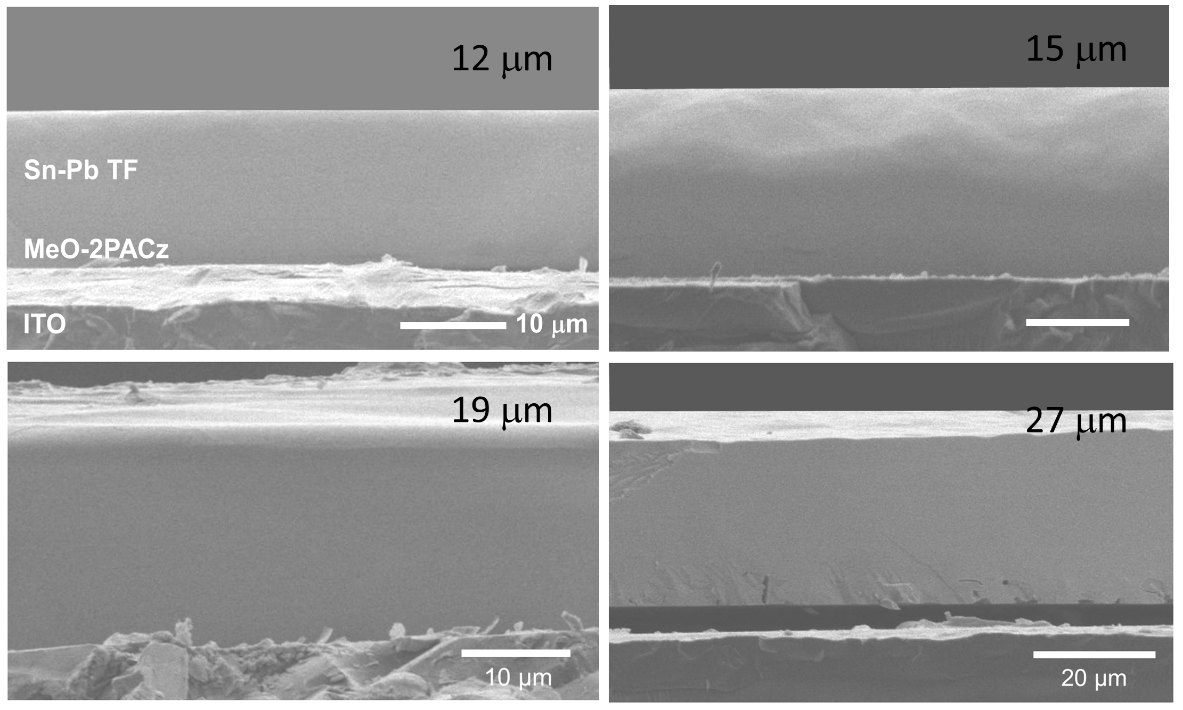
**

**Fig. S6** Cross-sectional SEM images of Sn–Pb single-crystal thin films (SCTFs) with thicknesses of 12 μm, 15 μm, 19 μm, and 27 μm, corresponding to different growth durations under the spatially confined crystallization protocol


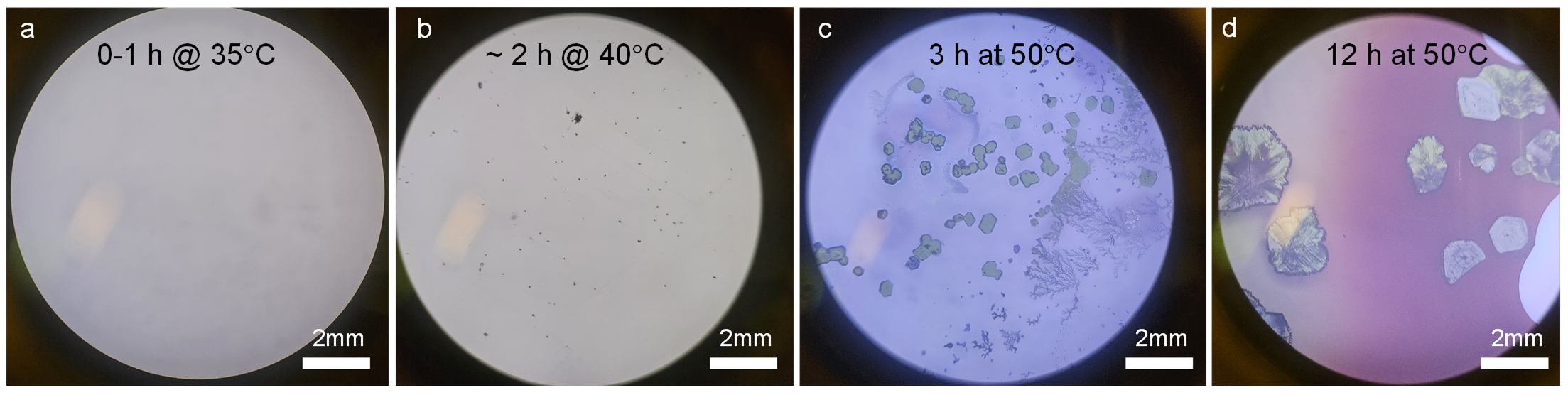


**Fig. S7** Thermal-profile-dependent nucleation and growth. (**a**) 35 °C, 0–1 h: no nuclei detected (ρ ≈ 0 mm⁻^2^). (**b**) 40 °C, ~2 h: nucleation onset (ρ ~0.80 mm⁻^2^). (**c**) 50 °C, 3 h: lateral growth/coarsening; medium size 650 ± 50 µm. (**d**) 50 °C, 12 h: millimeter-scale crystals (medium size 1400 ± 100 µm)


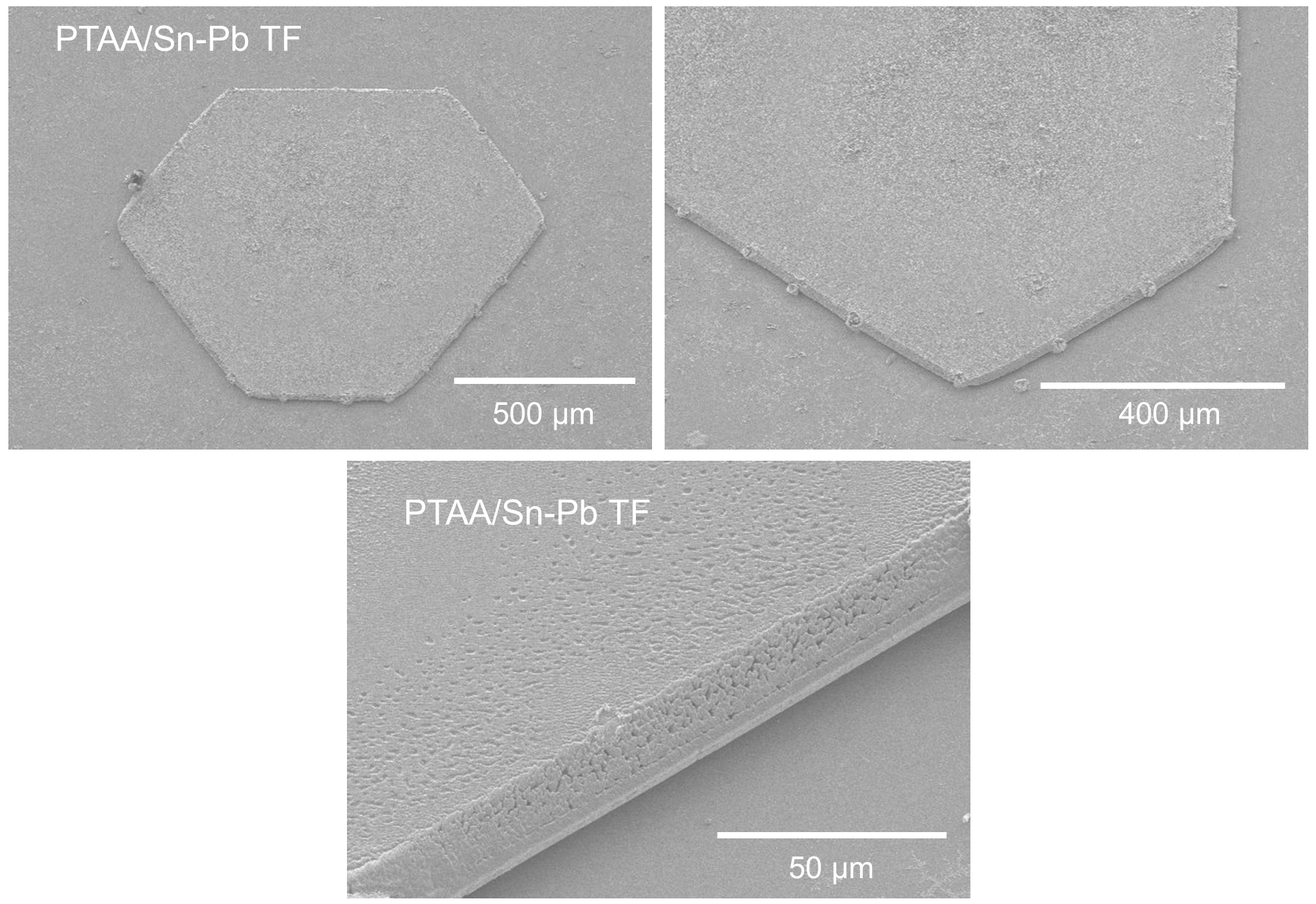


**Fig. S8** Top-view and cross-sectional SEM images of Sn–Pb single crystal films grown on hydrophobic PTAA/ITO substrates, showing poorly defined crystal edges and void-rich surface morphology


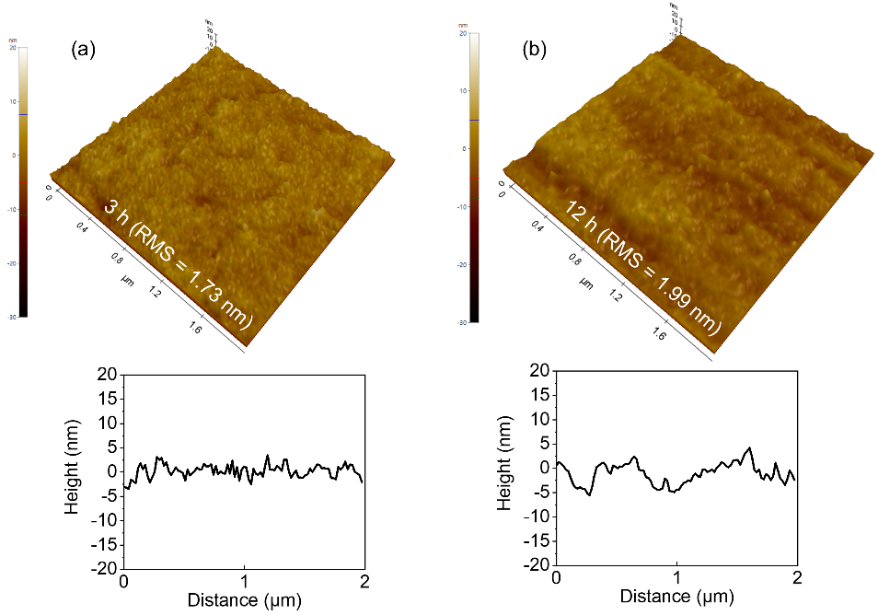


**Fig. S9** AFM of Sn–Pb single-crystal thin films at different growth durations: (**a**) 3 h, RMS = 1.73 nm, (**b**) 12 h, RMS = 1.99 nm. Bottom panels: representative line profiles over 2 µm scan lengths, showing sub-10 nm height variations. The films retain low nanometre-scale roughness with increased thickness, indicating smooth, uniform surfaces


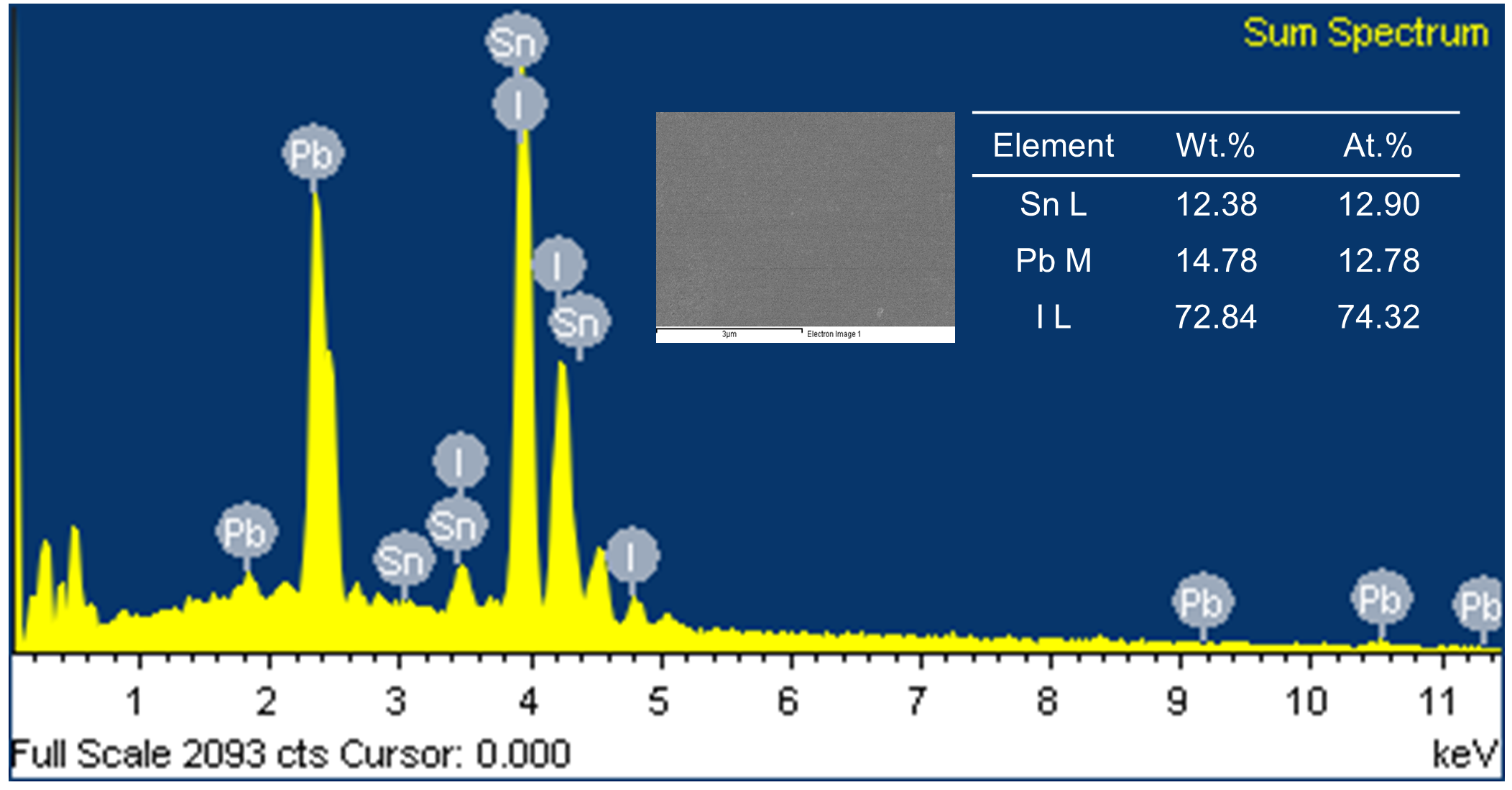


**Fig. S10** SEM-EDS analysis of as-grown micrometer-thick Sn–Pb SCTF, showing uniform stoichiometric composition of tin (Sn), lead (Pb), and iodine (I)

**
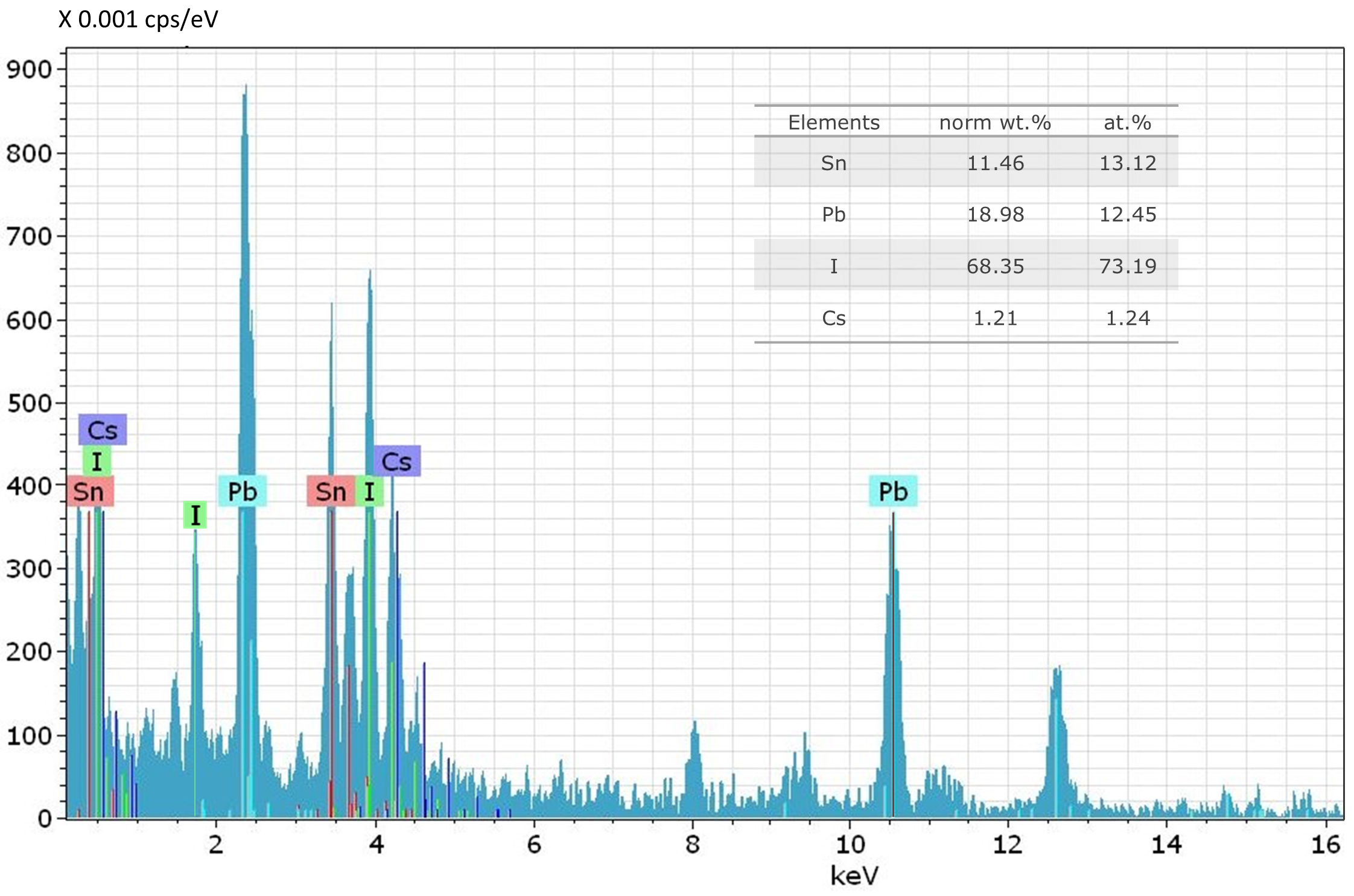
**

**Fig. S11** STEM–EDS spectra of Sn-Pb single-crystalline films. The inset table lists the quantified atomic percentages of Pb, Sn, I, and Cs confirming near-equimolar B-site ratios and stoichiometry close to the nominal precursor composition


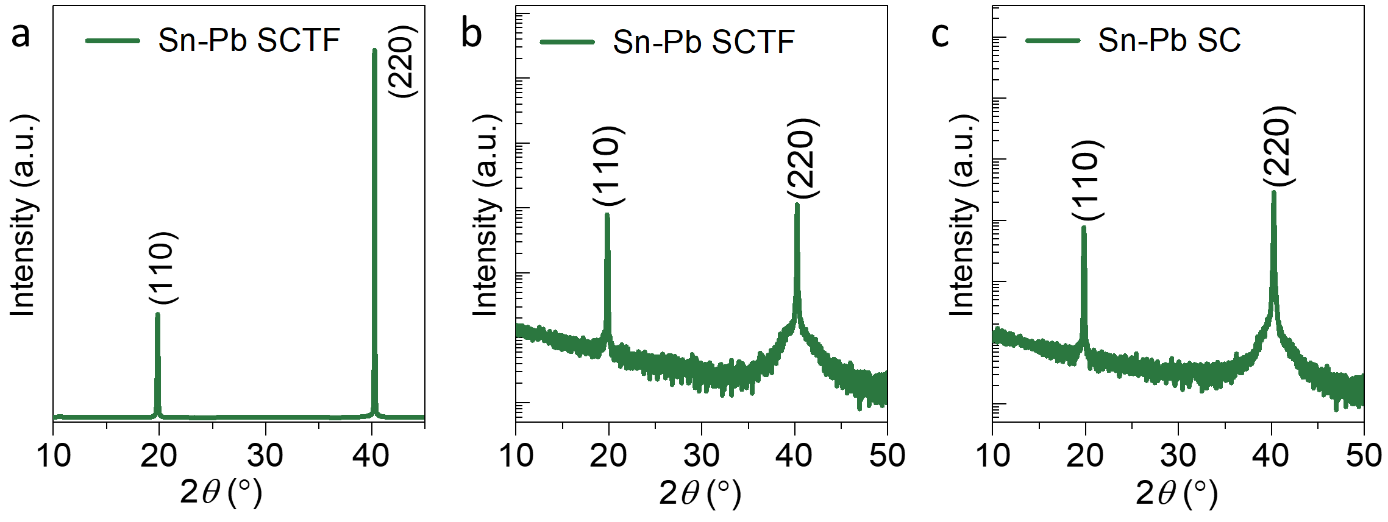


**Fig. S12** Single-crystal XRD pattern of Sn–Pb SCTF, along with a logarithmic (vertical axis) comparison of the diffraction patterns of as-grown Sn–Pb SCTF and bulk Sn–Pb single crystal

**
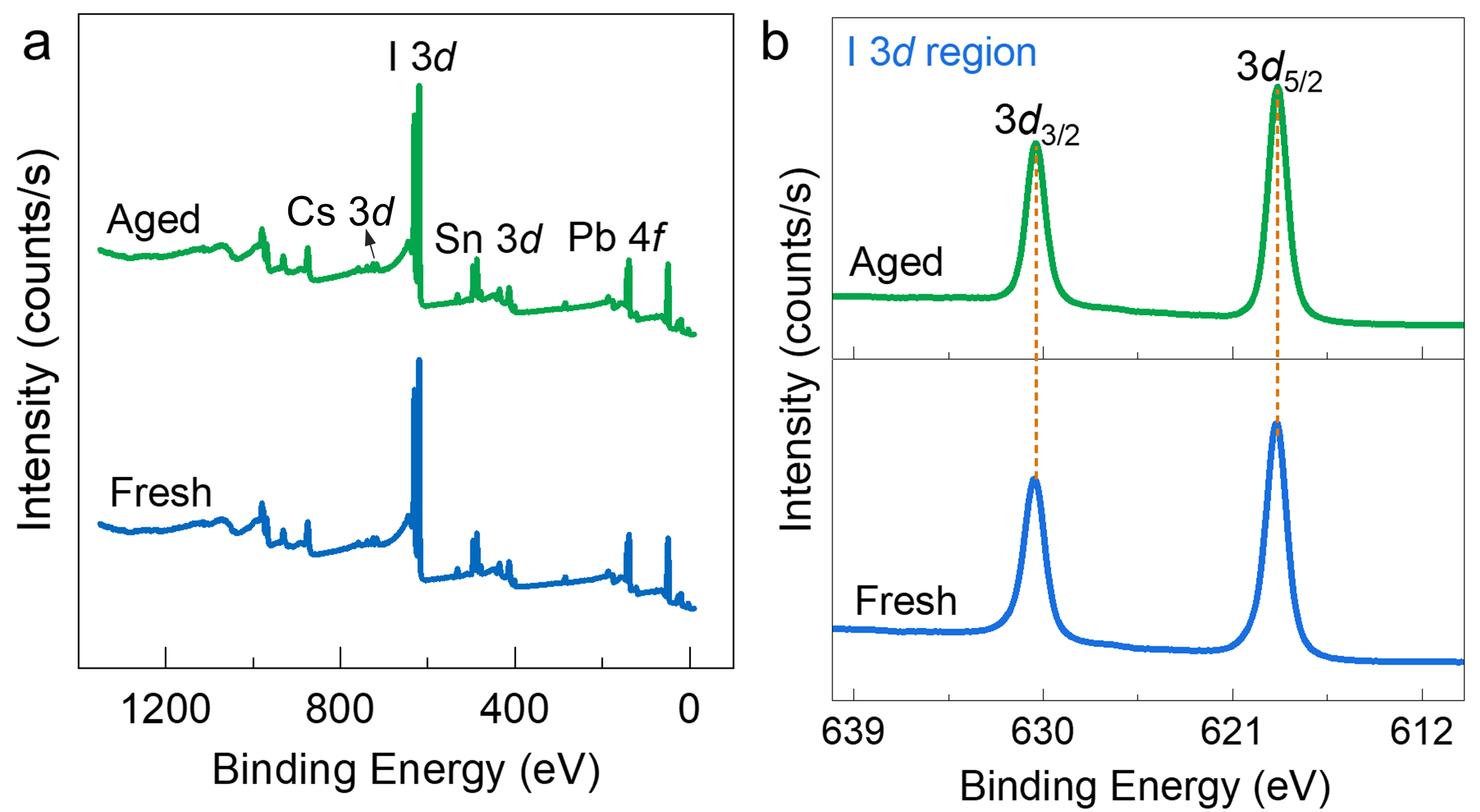
**

**Fig. S13** XPS survey spectrum and I 3*d* core-level spectra of Sn–Pb SCTF **a** before and **b** after ambient aging at 20% relative humidity (RH) for 100 h

**
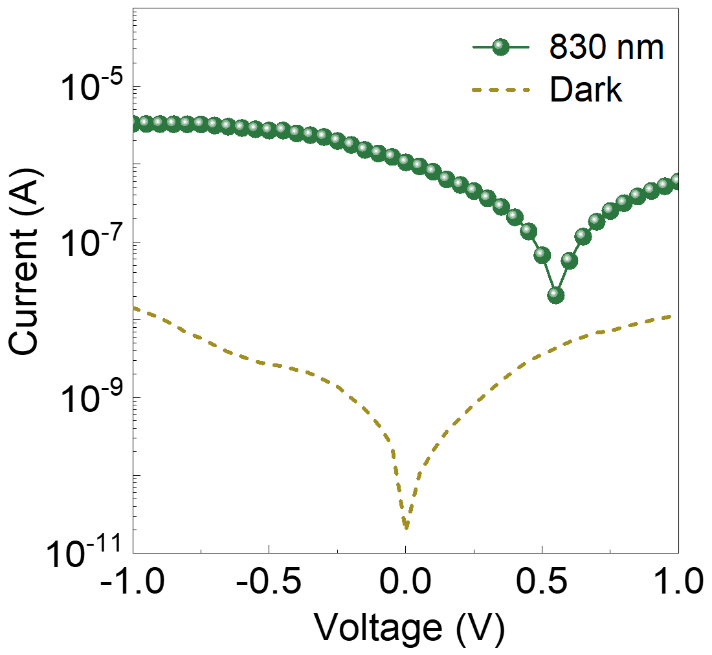
**

**Fig. S14** Dark and illuminated *I*-*V* curves of the Sn-Pb SCTF photodetector under 830 nm illumination at 65.1 mW cm^-2^

**
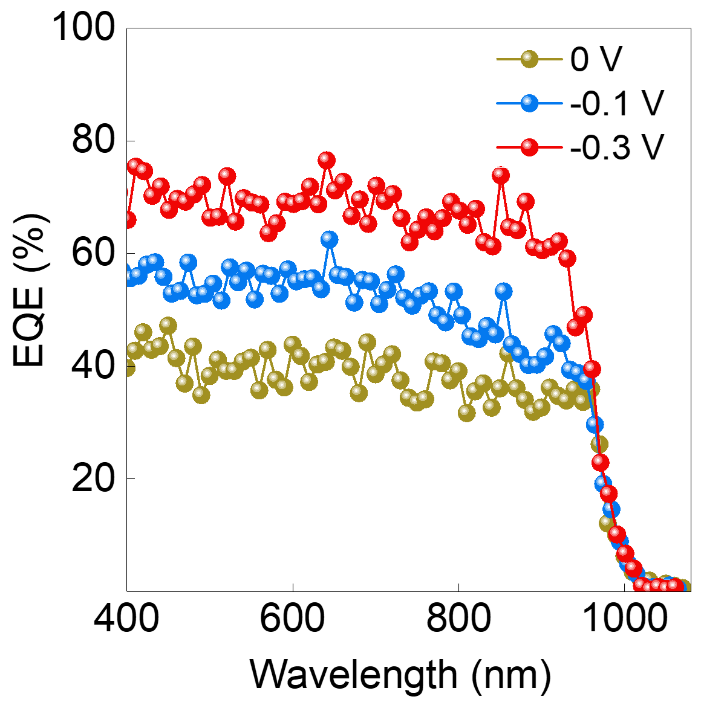
**

**Fig. S15** External quantum efficiency (EQE) spectra of Sn–Pb SCTF photodetectors under 830 nm illumination at applied biases of 0 V, –0.1 V, and –0.3 V

**
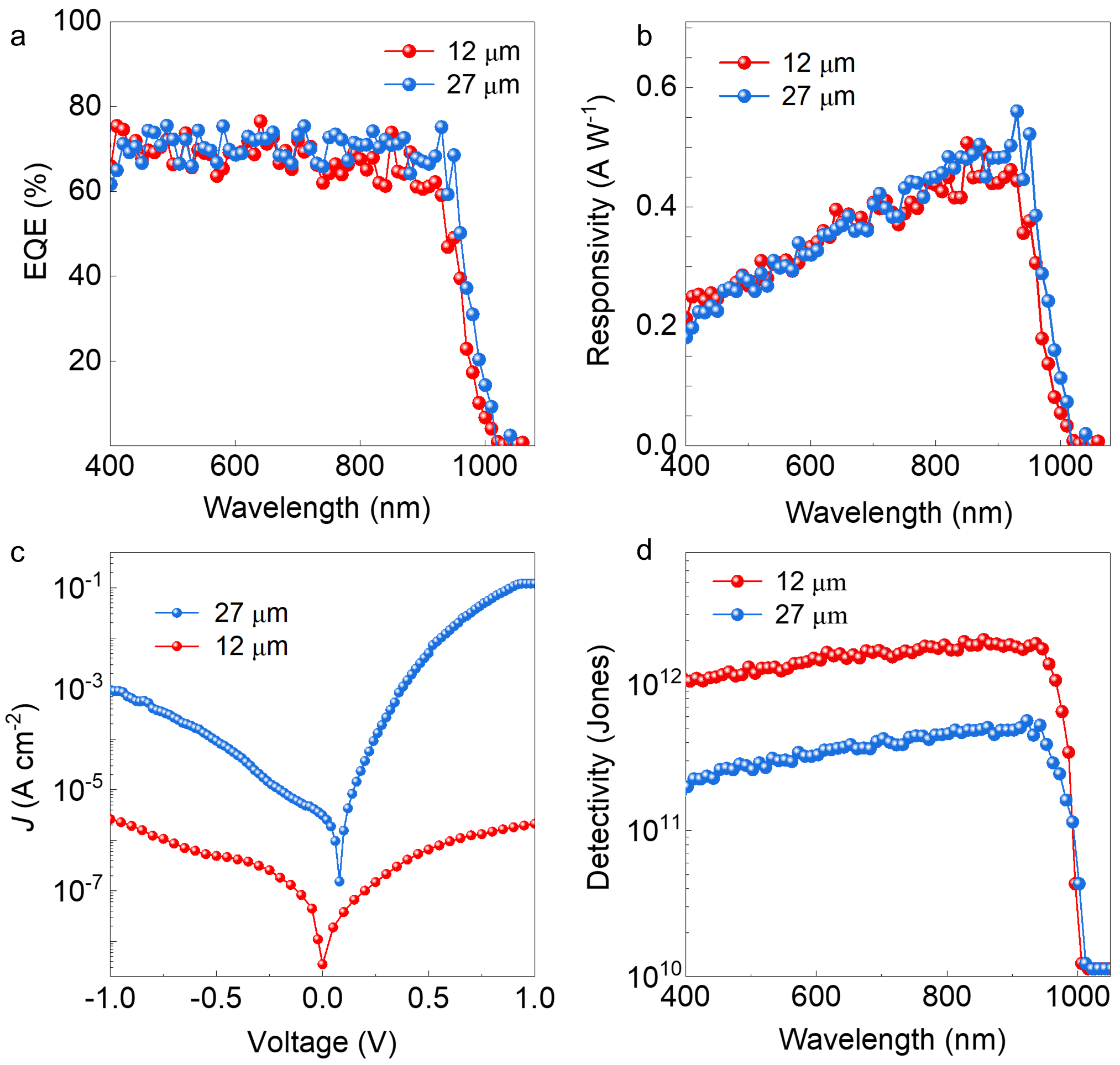
**

**Fig. S16** Thickness-dependent photodetector performance of Sn–Pb SCTFs. (**a**) EQE spectra, (**b**) Responsivity *R*(λ), (**c**) Dark current density *J*_d_, (**d**) Detectivity *D*(λ) calculated under shot-noise assumption for devices with 12 and 27 µm single-crystal films

**
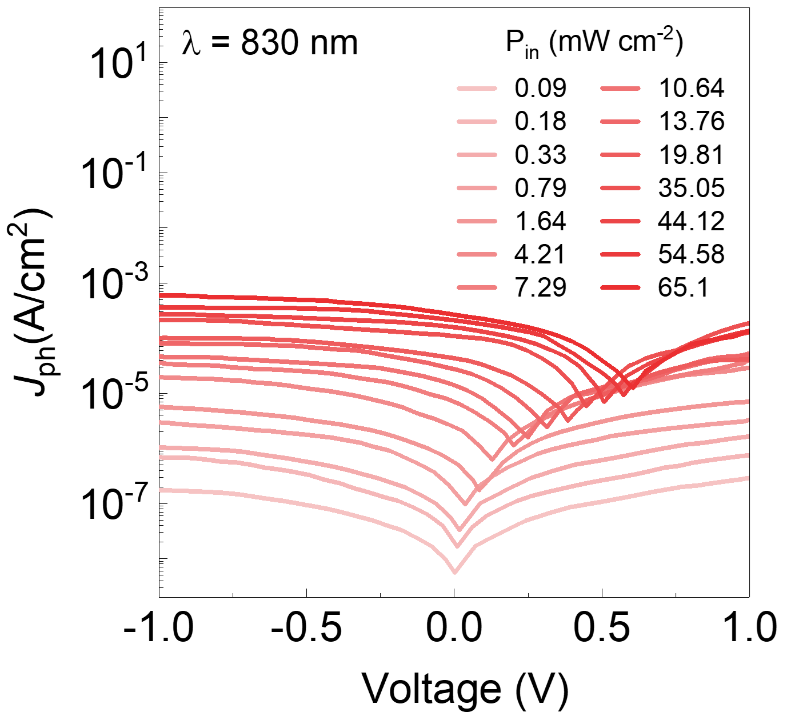
**

**Fig. S17** *J-V* curves of Sn-Pb single crystal photodetector measured under 830 nm laser illumination at incident light intensities ranging from 0.09 to 65.1 mW cm^-2^

**Table S1** Gutmann donor numbers [S1] and Dielectric constants of the cosolvents used for Sn-Pb perovskite single crystals growth process

| **Solvent** | **Donor Number (*D*_N_)** | **Dielectric constant (ε)** |
| --- | --- | --- |
| DMSO | 29.8 | 47 |
| gamma-Butyrolactone (GBL) | 18 | 35 |
| Acetonitrile (ACN) | 14.1 | 38 |
| Propylene carbonate (PC) | 15.1 | 62 |
| tetramethylene sulfone (TMS) | 14.7 | 40 |

**Table S2** Nucleation density and growth metrics from optical images

| **T (°C)** | **Time (h)** | **N(distinct nuclei in FOV)** | **FOV area (mm^2^)** | **ρ (mm^-2^)** | **Lateral size (µm)** | **Lateral growth rate (µm min^-1^)** |
| --- | --- | --- | --- | --- | --- | --- |
| 35 | 0-1 | 0 | 85 | 0 | - | - |
| 40 | 2 | 68 ± 5 | 85 | 0.80±0.06 | ~50±20 | - |
| 50 | 3 | n/a (growth stage) | 85 | n/a | ~650±120 | 10 |
| 50 | 12 | n/a (coarsened domains) | 85 | n/a | ~1400±250 | 1.4 |

Nucleation density ρ=N/A (mm⁻^2^), where N is the count of distinct nuclei inside the FOV. Growth rate as v =ΔL/Δt, with Δt taken over the indicated interval at 50 °C. ρ is reported only at the nucleation stage (2 h, 40 °C). At 3 h and 12 h, nuclei have grown, impinged and coarsened; counting features then reflects domains, not the original nucleation events, so we report sizes and growth rates instead. The FOV was constant (A = 85 mm^2^) for all images, calibrated from the 2 mm scale bar.

**Table S3** Thickness of Sn–Pb SCTFs as a function of growth time under confined ITC (precursor concentration = 1.2 M; substrate gap = ~100 µm). Thicknesses were measured from cross-sectional SEM at 60 °C, while lateral sizes were estimated from optical microscopy at 50 °C. Growth rates are calculated from linear fits of thickness versus time

| **Growth Time (h)** | **Thickness (µm, SEM@50 °C)** | **Thickness growth rate (µm h^-1^)** | **Lateral size (mm, optical image @50 °C)** |
| --- | --- | --- | --- |
| 3 | ~12 | ~1.3 | ~0.6 |
| 6 | ~15 | ~1.4 | - |
| 9 | ~19 | ~1.4 | - |
| 12 | ~27 | ~1.5 | ~1.4 |

**A-site (FA/MA) estimation by Vegard’s law**

We estimate the A-site ratio from the lattice parameter using previously established Vegard calibration for FA–MA–Cs perovskites in the cubic window [S2],

For FA*x*MA*y*Cs*z* with *x* + *y* + *z* = 1), the lattice parameter follows:

a = *x*a_FA_ + *y*a_MA_+ *z*a_Cs_, *y*=1−*x−z* (S1)

Solving for *x*(FA) gives,

$x=\frac{\left( aFA-MA-Cs \right)-\left( a\mathrm{MA} \right)-z\left( aCs-a\mathrm{MA} \right)}{(aFA-aMA)} y=1-x-z (S2)$

We used established FA–MA–Cs constants (a_FA_ (6.321 Å), a_MA_ (6.255 Å)_,_ and a_Cs_ (6.207 Å) [S3]. Cs fraction *z* = 0.050 (TEM/EDS), lattice parameter a= 6.291 Å from XRD gives A-site composition is FA_0.58_MA_0.37_Cs_0.05_. We use this Vegard result as a consistency check for the A-site composition, complementing direct B- and X-site analyses.

**Table S4** Carrier lifetime of Sn-Pb TF fitted by ExpDecay3 function in origin software and the average lifetime (τ_ave_) was calculated using the weighted average method [S4], as follows:

τ = (A_1_τ_1_+A_2_τ_2_+A_3_τ_3_)/(A_1_+A_2_+A_2_)

| **A_1_ [%]** | **τ_1_ [ns]** | **A_2_ [%]** | **τ_2_ [ns]** | **A_3_ [%]** | **τ_3_ [ns]** | **τ_avg_ [ns]** |
| --- | --- | --- | --- | --- | --- | --- |
| 12.91 | 7.73±0.06 | 25.29 | 54.72±1.03 | 61.80 | 277.17±3.09 | 186 |

**Table S5** Atomic percentage of relevant elements in fresh and aged Sn-Pb SCTF obtained from XPS analysis. Elemental ratios were derived from [Sn]/([Sn]+[Pb], [Pb]/([Sn]+[Pb], [I]/([Sn]+[Pb] and Cs/(Sn+Pb)

|  | **Name** | **Start BE** | **Peak BE** | **End BE** | **Atomic %** |
| --- | --- | --- | --- | --- | --- |
| Fresh | C1*s* | 292.41 | 284.73 | 280.86 | 15.30 |
|  | I3*d* | 635.17 | 618.92 | 612.54 | 62.93 |
|  | Pb4*f* | 147.58 | 138.07 | 134.13 | 10.95 |
|  | Sn3*d* | 499.34 | 486.82 | 481.78 | 10.82 |
|  | Cs3*d* | 727.95 | 724.64 | 720.46 | 1.26 |
|  | C1*s* | 292.98 | 284.83 | 281.98 | 15.18 |
|  | I3*d* | 635.49 | 618.88 | 612.79 | 62.97 |
| Aged | Pb4*f* | 147.65 | 138.16 | 134.48 | 10.97 |
|  | Sn3*d* | 499.59 | 486.85 | 481.94 | 10.88 |
|  | Cs3*d* | 727.94 | 724.67 | 720.48 | 1.23 |

**SCLC – Trap Density Calculation:**

The trap densities can be calculated using this equation,

$n_{traps}={2\varepsilon_{0}\varepsilon_{r}V_{TFL}}/{{ed}^{2}}$(S3)

(where ε_0_ represents the vacuum permittivity (8.854 × 10^-14^ F/cm^-1^); ε_r_ is the relative dielectric constant of perovskite (28.8); *e* is the elementary charge (1.602 × 10^-19^ coulombs); and *d* is the crystal thickness (~12 μm) [S5]. According to the literature, two relative dielectric constants were used for mixed Sn-Pb triple cation perovskites: 28.8 and 48.5 [S6–S8]. In both cases, trap densities of as-grown Sn-Pb SCTF is in the range of 10^12^ cm^-3^.

**Trap-free mobility (Mott–Gurney fit)**

The hole mobility (µ) was extracted from the trap-free SCLC (child) region using the Mott–Gurney relation (Eq. S4):

$J_{SCLC}\boldsymbol{=}\frac{9\varepsilon\varepsilon_{0}\mu V^{2}}{8L^{3}} (S4)$

Here L = 12 µm = 1.2 × 10^-3^ cm, A = 0.0049 cm^2^, ε_0_ = 8.854 × 10^-14^ F/cm^-1^, ε_r_ = 28.8. The hole mobility of 2.18 ± 0.06 cm^2^ V^−1^ s^−1^ was derived from the curve fitting of child region (Fig. 3d).

**Order-of-magnitude transit-time**

We estimate the characteristic collection time for a single-crystal slab of thickness L from the lowest-order diffusion mode:

τ_diff_ ≈ L^2^/π^2^D, where D = $\mu\frac{k_{B}T}{q} (S5)$

with L = 12 µm = 1.2 × 10^-3^ cm and *k*_B_T/q ≈ 0.0259 V (300 K), and using the SCLC-extracted hole mobility (2.18 ± 0.06 cm^2^ V^−1^ s^−1^):

D = 0.05646 cm^2^ s^−1^, τ_diff_ = 2.6 × 10^-6^ s (2.6 µs).

An equivalent drift–diffusion scaling τ ≈ L^2^/(μV_eff​_) gives 10^2^-µs magnitudes for small effective fields (V_eff_ ≈ 3.6×10^-3^ V from scaling equation).

The noise-equivalent power (NEP) was determined as:

$$\mathrm{NEP} \left( f \right)=\frac{\sqrt{S_{i}\left( f \right)}}{R\left( \lambda\right)} (S6)$$

Where *S_i_(f)* is the measured current noise spectral density and R(λ) is the responsivity.

The specific detectivity was then calculated as:

$$D*\left( \lambda,f \right)=\frac{\sqrt{A}}{NEP\left( f \right)}= \frac{R\left( \lambda\right)\sqrt{A}}{i_{n}\left( f \right)} (S7)$$

**Table S6** Comparison of composition, growth method, temperature, single crystal thicknesses, and trap-state density of Sn or Sn-Pb-based perovskite single crystals reported in previous studies

| **Composition** | **Methods** | **Temperature and Time** | **Thickness** | **Trap density**  **(cm^-3^)** | **Refs.** |
| --- | --- | --- | --- | --- | --- |
| CH_3_NH_3_SnI_3_ | Synthetic method with reductants | 100 °C, 12 h | 110 μm | - | [S9] |
| CH_3_NH_3_SnI_3_ | Cooling crystallization | 90 °C, 48 h | 6 mm | - | [S10] |
| CH_3_NH_3_SnI_3_ | Inverse temperature crystallization (ITC) | 70 °C, 3 h | 20 μm | - | [S11] |
| CH_3_NH_3_SnI_3_ and FASnI_3_ | Top-seeded crystal growth | 75 °C, 730 h | 5 mm | 10^12^ and 10^13^ | [S12] |
| FASnI_3_ | Temperature-lowering method in HBr acid | 75 °C, 72 h | - | - | [S13] |
| CsSnI_3_ | Modified vertical  Bridgman technique | 550 °C, 1 h | - | - | [S14] |
| Cs_2_SnCl_6−_*_x_*Br*_x_* | Hydrothermal method | 180 °C, 36 h | 1.3 mm | - | [S15] |
| Graded MAPb_0.5+x_Sn_0.5-x_I_3_ | Lithography-assisted epitaxial-growth | 160 °C, 5 h | 10 μm | 3.34×10^12^ - 1.77×10^14^ | [S16] |
| MAPb_0.5_Sn_0.5_I_3_ | Conventional ITC method | 95 °C, 6 h | 15 μm | 6.948×10^13^ | [S17] |
| Polycrystals of  FA_0.55_MA_0.4_Cs_0.05_Sn_0.5_Pb_0.5_I_3_ | Spin-coating method | 100 °C, 0.5 h | 850 nm | 3.05×10^15^ | [S8] |
| SCTF of  FA_0.55_MA_0.4_Cs_0.05_Sn_0.5_Pb_0.5_I_3_ | Cosolvent ITC method | 45 °C, 6 h | 12 μm | 3.98×10^12^ | This  work |

**Table S7** Performance comparison of our optimized Sn–Pb perovskite single-crystal photodetector with commercial and prior NIR perovskite photodetectors, listing peak wavelength, peak responsivity, specific detectivity (*D**) under noise, detectivity (*D*) under shot-noise limited, and LDR. Device metrics were measured at 830 nm (65.1 mW cm⁻^2^) on 0.0049 cm^2^ devices at 0, −0.1, and −0.3 V in ambient air (22–25 °C, ~55% RH; unencapsulated)

| Sn-Pb mixed  perovskites | λ_peak_ (nm) | R_peak_  (AW^-1^) | *D** and D  (Jones) | LDR (dB) | Refs. |
| --- | --- | --- | --- | --- | --- |
| FA_0.55_MA_0.4_Cs_0.05_  Sn_0.5_Pb_0.5_I_3_ (SCTF) | 900 | 0.51 | 1.26 × 10^10^ (*D**) | 96 | This work |
| (MAPbI_3_)_0.2_  (FASnI_3_)_0.8_ (SC) | 1064 | 0.24 | 1.17 × 10^12^ (*D)* | - | [S18] |
| (FASnI_3_)_0.1_  (MAPbI_3_)_0.9_ (SC) | 808 | 0.53 | 7.09 × 10^10^  (*D**) | 87 | [S19] |
| MAPb_0.5_Sn_0.5_I_3_ (SC) | 905 | 0.0016 | 3.08 × 10^10^  (*D**) | - | [S17] |
| MAPb_0.76_Sn_0.24_I_3_ (SC) | 920 | - | 1.19 × 10^10^  (*D**) | - | [S20] |
| MA_0.5_FA_0.5_Pb_0.5_Sn_0.5_I_3_ (film) | 900 | 0.1 | 10^12^  (*D)* | - | [S21] |
| Cs_0.05_MA_0.45_FA_0.5_  Sn_0.5_Pb_0.5_I_3_ (film) | 900 | 0.18 | 10^12^  (*D)* | 83.6 | [S22] |
| FA_0.85_Cs_0.15_Sn_0.5_Pb_0.5_I_3_ (Film) | 910 | 0.5 | 1.04 × 10^12^  (*D)* | - | [S23] |
| FA_0.7_MA_0.3_Sn_0.5_Pb_0.5_I_3_ (film) | 930 | 0.40 | 7.5 × 10^11^  (*D)* | - | [S24] |
| (FASnI_3_)_0.6_  (MAPbI_3_)_0.4_ (film) | 900 | 0.40 | 1.1 × 10^12^  (*D)* | - | [S25] |
| Si photodetectors | 950 | 0.35 | - | - | [S26] |
| FA_0.7_MA_0.3_Pb_0.5_Sn_0.5_I_3_ (film) | 830 | 0.49 | 1.20 × 10^12^  (*D*)* | 174 | [S27] |
| MA_0.5_FA_0.5_Pb_0.5_Sn_0.5_I_3_ (film) | 940 | 0.10 | 4.2 × 10^12^  (*D)* | - | [S28] |

**Table S8** Operational-stability parameters for polycrystalline Sn–Pb devices with composition, environment, illumination/modulation, encapsulation, and retention/failure metrics as reported

| **Composition** | **Temperature/ Atmosphere/**  **Encapsulation** | **Illumination (λ, intensity)**  **/modulation** | **Stability metric** | **Refs.** |
| --- | --- | --- | --- | --- |
| FA_0.55_MA_0.4_Cs_0.05_  Sn_0.5_Pb_0.5_I_3_ (SCTF) | ~22-25°C; 55 RH%.  unencapsulated | 830 nm; 65.1 mW cm⁻²/10 Hz | ≥95% photocurrent retained after 25,000 cycles | This work |
| Cs_0.15_FA_0.85_Pb_0.5_Sn_0.5_I_3_  (film) | ~RT; Nil.  unencapsulated | 750 nm; Nil | ~88% photocurrent retained after 552 h; ~30% after 984 h | [S29] |
| (FASnI_3_)_0.6_(MAPbI_3_)_0.4_ (film) | ~RT; Nil.  unencapsulated | 900 nm; 11.4 µW cm⁻² | ~100% after 50 cycles/1000 s | [S30] |
| FASnI_3_)_0.6_(MAPbI_3_)_0.4_ (film) | ~RT; air  unencapsulated | 810 nm; 360 mW cm⁻² | ~95% photocurrent retained after 700 min | [S31] |
| FA_0.66_MA_0.34_Pb_0.5_Sn_0.5_I_3_(film) | ~RT; N_2_  unencapsulated | 930 nm; 65.1 mW cm⁻² | ~95% photocurrent retained after 4200 s | [S32] |
| (FASnI_3_)_0.6_(MAPbI_3_)_0.4_ (film) | ~RT; air  unencapsulated | 650 nm; 2 mW cm⁻² | ~80% photocurrent retained after 200 cycles | [S33] |

**Supplementary References**

1. J.C. Hamill Jr, J. Schwartz, Y.-L. Loo, Influence of solvent coordination on hybrid organic–inorganic perovskite formation. ACS Energy Lett. **3**(1), 92–97 (2018). <https://doi.org/10.1021/acsenergylett.7b01057>
2. N.J. Jeon, J.H. Noh, W.S. Yang, Y.C. Kim, S. Ryu et al., Compositional engineering of perovskite materials for high-performance solar cells. Nature **517**(7535), 476–480 (2015). <https://doi.org/10.1038/nature14133>
3. R.K. Gunasekaran, J. Jung, S.W. Yang, J. Yun, Y. Yun et al., High-throughput compositional mapping of triple-cation tin–lead perovskites for high-efficiency solar cells. InfoMat **5**(4), e12393 (2023). <https://doi.org/10.1002/inf2.12393>
4. T. Jinhui, S. Zhaoning, K.D. Hoe, C. Xihan, C. Cong et al., Carrier lifetimes of >1 μs in Sn-Pb perovskites enable efficient all-perovskite tandem solar cells. Science **364**(6439), 475-479 (2019). <https://doi.org/10.1126/science.aav7911>
5. R.K. Gunasekaran, J. Jung, S.W. Yang, J. Yun, Y. Yun et al., High-throughput compositional mapping of triple-cation tin–lead perovskites for high-efficiency solar cells. InfoMat **5**(4), e12393 (2023). <https://doi.org/10.1002/inf2.12393>
6. Y. Liu, Y. Zhang, X. Zhu, Z. Yang, W. Ke et al., Inch-sized high-quality perovskite single crystals by suppressing phase segregation for light-powered integrated circuits. Sci. Adv. **7**(7), eabc8844 (2021). <https://doi.org/10.1126/sciadv.abc8844>
7. S. Lee, M.Y. Woo, C. Kim, K.W. Kim, H. Lee et al., Buried interface modulation *via* PEDOT: PSS ionic exchange for the Sn-Pb mixed perovskite based solar cells. Chem. Eng. J. **479**, 147587 (2024). <https://doi.org/10.1016/j.cej.2023.147587>
8. R.K. Gunasekaran, J. Jung, S.W. Yang, D. Im, W.C. Choi et al., Regulating surface heterogeneity maximizes photovoltage and operational stability in tin–lead perovskite solar cells. ACS Energy Lett. **9**(1), 102–109 (2024). <https://doi.org/10.1021/acsenergylett.3c02402>
9. Z. Yao, Z. Yang, Y. Liu, W. Zhao, X. Zhang et al., Local temperature reduction induced crystallization of MASnI_3_ and achieving a direct wafer production. RSC Adv. **7**(61), 38155–38159 (2017). <https://doi.org/10.1039/C7RA07101A>
10. Y.Q. Huang, J. Su, Q.F. Li, D. Wang, L.H. Xu et al., Structure, optical and electrical properties of CH_3_NH_3_SnI3 single crystal. Phys. B Condens. Matter **563**, 107–112 (2019). <https://doi.org/10.1016/j.physb.2019.03.035>
11. Z. Yuan, J. Zhou, Y. Zhang, X. Ma, J. Wang et al., Growing MASnI_3_ perovskite single-crystal films by inverse temperature crystallization. J. Phys.: Condens. Matter **34**(14), 144009 (2022). <https://doi.org/10.1088/1361-648x/ac4c64>
12. Y. Dang, Y. Zhou, X. Liu, D. Ju, S. Xia et al., Formation of hybrid perovskite tin iodide single crystals by top-seeded solution growth. Angew. Chem. Int. Ed. **55**(10), 3447–3450 (2016). <https://doi.org/10.1002/anie.201511792>
13. L. He, H. Gu, X. Liu, P. Li, Y. Dang et al., Efficient anti-solvent-free spin-coated and printed Sn-perovskite solar cells with crystal-based precursor solutions. Matter **2**(1), 167–180 (2020). <https://doi.org/10.1016/j.matt.2019.10.006>
14. I. Chung, J.-H. Song, J. Im, J. Androulakis, C.D. Malliakas et al., CsSnI_3_: semiconductor or metal? high electrical conductivity and strong near-infrared photoluminescence from a single material. high hole mobility and phase-transitions. J. Am. Chem. Soc. **134**(20), 8579–8587 (2012). <https://doi.org/10.1021/ja301539s>
15. J. Zhou, J. Luo, X. Rong, P. Wei, M.S. Molokeev et al., Lead-free perovskite derivative Cs_2_SnCl_6−_*_x_*Brx single crystals for narrowband photodetectors. Adv. Opt. Mater. **7**(10), 1900139 (2019). <https://doi.org/10.1002/adom.201900139>
16. Y. Lei, Y. Chen, R. Zhang, Y. Li, Q. Yan et al., A fabrication process for flexible single-crystal perovskite devices. Nature **583**(7818), 790–795 (2020). <https://doi.org/10.1038/s41586-020-2526-z>
17. J. Wu, Y. Zhang, S. Yang, Z. Chen, W. Zhu, Thin MAPb_0.5_Sn_0.5_I_3_ perovskite single crystals for sensitive infrared light detection. Front Chem **9**, 1 (2022). <https://doi.org/10.3389/fchem.2021.821699>
18. Q. Li, S. Wang, C. Jia, H. Liu, X. Li, High-performance planar self-driven visible and near-infrared photodetector based on Pb-Sn perovskite single crystals. J. Phys. Chem. Lett. **14**(22), 5148–5154 (2023). <https://doi.org/10.1021/acs.jpclett.3c01083>
19. Z. Chang, Z. Lu, W. Deng, Y. Shi, Y. Sun et al., Narrow-bandgap Sn-Pb mixed perovskite single crystals for high-performance near-infrared photodetectors. Nanoscale **15**(10), 5053–5062 (2023). <https://doi.org/10.1039/d2nr05800f>
20. X. Xu, C.-C. Chueh, P. Jing, Z. Yang, X. Shi et al., High-performance near-IR photodetector using low-bandgap MA0.5FA0.5Pb_0.5_Sn_0.5_I_3_ perovskite. Adv. Funct. Mater. **27**(28), 1701053 (2017). <https://doi.org/10.1002/adfm.201701053>
21. W. Li, J. Chen, H. Lin, S. Zhou, G. Yan et al., The UV–vis-NIR broadband ultrafast flexible Sn-Pb perovskite photodetector for multispectral imaging to distinguish substance and foreign-body in biological tissues. Adv. Opt. Mater. **12**(2), 2301373 (2024). <https://doi.org/10.1002/adom.202301373>
22. J. Liu, Y. Chen, J. Zhou, J. Wang, Z. Chen et al., Filterless near-infrared narrowband photodetectors based on mixed metal perovskite single crystals. Adv. Opt. Mater. **12**(32), 2401544 (2024). <https://doi.org/10.1002/adom.202401544>
23. H. Li, Y. Gao, X. Hong, K. Ke, Z. Ye et al., Rational composition engineering for high-quality Pb–Sn photodetector toward sensitive near-infrared digital imaging arrays. InfoMat **7**(1), e12615 (2025). <https://doi.org/10.1002/inf2.12615>
24. W. Wang, D. Zhao, F. Zhang, L. Li, M. Du et al., Highly sensitive low-bandgap perovskite photodetectors with response from ultraviolet to the near-infrared region. Adv. Funct. Mater. **27**(42), 1703953 (2017). <https://doi.org/10.1002/adfm.201703953>
25. R. Ollearo, J. Wang, M.J. Dyson, C.H.L. Weijtens, M. Fattori et al., Ultralow dark current in near-infrared perovskite photodiodes by reducing charge injection and interfacial charge generation. Nat. Commun. **12**(1), 7277 (2021). <https://doi.org/10.1038/s41467-021-27565-1>
26. C. Fuentes-Hernandez, W.-F. Chou, T.M. Khan, L. Diniz, J. Lukens et al., Large-area low-noise flexible organic photodiodes for detecting faint visible light. Science **370**(6517), 698–701 (2020). <https://doi.org/10.1126/science.aba2624>
27. H. Li, Y. Gao, X. Hong, K. Ke, Z. Ye, S. Zhang, K. Shi, Z. Peng, H. Yan, M.-C. Tang, Y. Yao, B. Z. Tang, G. Wei, and F. Kang, InfoMat 7, e12615 (2024). <https://doi.org/10.1002/inf2.12615>
28. H. Liu, L. Zhu, H. Zhang, X. He, F. Yan et al., Realizing high-detectivity near-infrared photodetectors in tin–lead perovskites by double-sided surface-preferred distribution of multifunctional tin thiocyanate additive. ACS Energy Lett. **8**(1), 577–589 (2023). <https://doi.org/10.1021/acsenergylett.2c02055>
29. Y. Ma, L. Shan, Y. Ying, L. Shen, Y. Fu et al., Day-Night imaging without Infrared Cutfilter removal based on metal-gradient perovskite single crystal photodetector. Nat. Commun. **15**(1), 7516 (2024). <https://doi.org/10.1038/s41467-024-51762-3>
30. W. Wang, D. Zhao, F. Zhang, L. Li, M. Du et al., Highly sensitive low-bandgap perovskite photodetectors with response from ultraviolet to the near-infrared region. Adv. Funct. Mater. **27**(42), 1703953 (2017). <https://doi.org/10.1002/adfm.201703953>
31. K.-R. Yun, T.-Y. Seong, Achieving high-performance photodetectors through defect passivation enabled by additive engineering of Pb–Sn mixed perovskites. ACS Appl. Electron. Mater. **7**(16), 7885–7895 (2025). <https://doi.org/10.1021/acsaelm.5c01284>
